# ROEP: A Robotics-Oriented Evaluation Protocol for Deployment-Facing Vision–Language–Action Manipulation Policies

**DOI:** 10.3390/s26154757

**Published:** 2026-07-27

**Authors:** Sangwoo Han, Hyunguk Choi

**Affiliations:** Department of Artificial Intelligence Software, Cheongju University, Cheongju 28503, Republic of Korea; soonawg@cju.ac.kr

**Keywords:** vision–language–action, robotic manipulation, deployment-oriented evaluation, multimodal sensing, robustness evaluation

## Abstract

Vision–Language–Action (VLA) policies are increasingly evaluated on language-conditioned robotic manipulation benchmarks, but success rate alone often obscures runtime-interface alignment, the repeatability of observation-degradation effects, and the recovery-relevant semantics of failures. This study proposes ROEP (Robotics-Oriented Evaluation Protocol), a VLA-targeted deployment-oriented evaluation protocol that converts rollout outcomes into claim-level evidence for closed-loop robotic manipulation. ROEP first verifies clean-condition evaluability and runtime fidelity of the sensor-to-action execution interface, then evaluates controlled visual perturbations, repeated-run reference variability, timeout-dominant failures, and recovery/shield support. We apply ROEP to OpenVLA, X-VLA, and VLA-Adapter on eight LIBERO Goal and Object tasks, producing a 24-row evaluation matrix with complete clean and medium-perturbation evidence. Under the evaluated LIBERO simulation setting, the results show that runtime-interface fidelity and checkpoint provenance are important for interpreting X-VLA and OpenVLA Object results, while VLA-Adapter maintains strong Goal-suite performance but exhibits a substantial Object-suite clean-to-perturbation drop dominated by timeout termination. ROEP therefore clarifies which deployment-relevant claims are supported, withheld, or insufficiently evidenced by the available rollouts without certifying open-world deployment safety or recovery success. The protocol complements existing VLA benchmarks by reporting success rate together with runtime fidelity, repeatability, failure semantics, and recovery/shield support evidence.

## 1. Introduction

Vision and multimodal sensing are central to intelligent robotic systems that perceive their surrounding environments and determine appropriate actions. In healthcare, manufacturing, logistics, and service robotics, a robot must go beyond merely detecting objects. It must interpret user intent, select the manipulation action required by the given scene, and execute the task reliably until completion. In this context, robotic manipulation research has moved toward combining cameras, depth sensors, proprioceptive signals, and natural-language instructions. Recently, Vision–Language–Action (VLA) policies, which connect visual observations and language instructions directly to action generation, have attracted growing attention  [1,2,3,4]. Furthermore, learning for control has emerged as a significant trend in robotics, offering flexible and adaptive solutions for complex systems [5].

A VLA policy receives an image and a natural-language command as input and generates actions to complete the task. RT-2 introduced the idea of directly connecting vision-language models to robotic control, while Open X-Embodiment and OpenVLA expanded the basis for large-scale robot demonstrations and open-source VLA research [2,3,4]. Open-source generalist robot policies such as Octo further show that diverse sensor/action interfaces and downstream fine-tuning conditions must be considered together, suggesting that VLA evaluation should include not only model architecture but also the execution interface and task condition [6]. More recent models such as X-VLA and VLA-Adapter emphasize cross-embodiment adaptation, efficient action bridging, and smaller-backbone VLA design, extending the practical usability of VLA policies [7,8]. Together, these studies suggest that VLA policies should be evaluated as closed-loop robotic systems that integrate sensing, language understanding, and action execution.

A key challenge is that current evaluation practices often fail to answer the questions that must be addressed prior to deployment. In robotic manipulation research, task success rate on benchmark tasks remains the most widely used metric. Although success rate is important because it measures basic manipulation capability, it is not sufficient on its own. Recent work on manipulation generalization and VLA robustness has reported that nominal benchmark performance can be highly sensitive to changes in texture, lighting, camera pose, object layout, robot initial state, and sensor noise [9,10,11]. For example, two policies may achieve the same success rate, yet one may experience a sharp performance drop when the camera image is slightly degraded, while the other may exhibit poor repeatability across repeated trials under the same condition. In other cases, the model itself may not be the primary cause of failure; instead, failures may arise because the checkpoint, image preprocessing, action scaling, or control mode does not match the runtime interface for which the model was designed. Therefore, VLA policy evaluation should examine not only whether a task succeeds, but also under what conditions, with what degree of repeatability, and through which execution interface the result is obtained.

Existing studies offer useful starting points for addressing this limitation. LIBERO provides standardized task suites for language-conditioned robotic manipulation, allowing different policies to be compared under shared task conditions [12]. Related work in robotics and sensing systems has also treated evaluation protocols and benchmarking frameworks as research contributions in their own right. For instance, studies on LLM-based robot navigation have proposed benchmarking frameworks that jointly evaluate latency, parsing accuracy, path quality, and task success rate, while work on intelligent medical radar has introduced a protocol that organizes evaluation targets, experimental conditions, reference measurements, and metrics in a systematic manner [13,14]. In addition, studies on vision-system evaluation for collaborative-robot applications and dynamic validation of multi-sensor mobile robots indicate that sensing-system performance should be assessed across diverse operating conditions, with attention to repeatability and system fidelity, rather than reduced to a single accuracy metric [15,16].

Despite these advances, deployment-oriented evaluation protocols for VLA manipulation policies remain insufficiently established. A common approach is to regard benchmark success rate as the primary evidence of policy quality and to interpret robustness mainly through the magnitude of success-rate degradation under perturbations. While this approach is useful for basic model comparison, it leaves the underlying causes of performance degradation unresolved. In contrast, a more conservative approach first disentangles several factors before interpreting perturbation results: whether execution under clean conditions is sufficiently reliable, how much outcomes vary across repeated trials under the same condition, and whether failures arise from task incompletion, execution-interface mismatch, timeout, or the absence of recovery support. This paper adopts the latter approach.

To address this gap, we propose ROEP (Robotics-Oriented Evaluation Protocol), an evaluation protocol for assessing which deployment-relevant claims can be supported by existing rollout results of robotic manipulation VLA policies. ROEP is VLA-targeted: it keeps the task suite and sensor hardware fixed while evaluating how a trained VLA policy behaves when closed-loop sensing-to-action execution is filtered through clean admission, controlled observation degradation, repeated-run reference variability, and runtime-interface checks. Specifically, ROEP first verifies whether each model-task pair satisfies a minimum clean-condition evaluability floor before downstream analysis is opened. It then evaluates outcomes under visual perturbations, estimates natural variability across repeated executions under the same condition, and checks whether the checkpoint and runtime interface are properly aligned with the model’s intended execution interface. Finally, ROEP incorporates failure patterns and recovery/shield support evidence, which are not captured by success rate alone, to translate raw rollout outcomes into claim-level decisions.

ROEP aims to constrain the scope of claims to the evidence observed, rather than to produce higher scores. Rows lacking reliable clean-condition execution have downstream claim interpretation withheld, rather than being overstated as perturbation-robustness failures. Even when both clean-execution and perturbation evidence are available, recovery-facing claims are withheld separately if repeated-execution variability, runtime fidelity, or recovery/shield support is insufficient. Thus, ROEP outputs not merely a success-rate table, but a decision table that specifies, for each model-task pair and condition, which claims are supported and which are withheld.

In this study, we evaluate OpenVLA, X-VLA, and VLA-Adapter on the LIBERO Goal and Object task suites, analyzing 24 model-suite-task rows. Our results show that success-only tables and protocol-constrained interpretations can diverge substantially. In particular, runtime-interface fidelity and checkpoint provenance affect the interpretability of rollout results, while perturbation conditions reveal interpretive factors beyond success-rate degradation, including timeout termination and limitations in recovery/shield support. These findings indicate that evaluations of VLA manipulation policies must report success rate alongside runtime fidelity, repeatability, failure semantics, and recovery/shield support.

The central contribution of ROEP is not another success-rate table, but a claim-support protocol for VLA manipulation evaluation. Instead of asking only whether a policy succeeds under a nominal benchmark condition, ROEP asks whether the evidence behind a model-task row is sufficient to support a deployment-facing interpretation. This study makes four contributions. First, it defines a VLA-targeted evaluation flow that combines clean admission, controlled observation degradation, repeated-run reference variability, runtime/interface fidelity, failure semantics, and recovery/shield support. Second, it formalizes a conservative claim-status rule that prevents low clean support, runtime mismatch, or insufficient recovery/shield evidence from being misreported as perturbation robustness. Third, it demonstrates the practical applicability of ROEP through a 24-row LIBERO Goal/Object evaluation of OpenVLA, X-VLA, and VLA-Adapter. Fourth, it shows that sensor-to-action runtime fidelity and timeout-dominant completion failure can change how VLA rollout results should be interpreted under controlled visual perturbation.

## 2. Related Work

### 2.1. Vision and Multimodal Sensing for Robotic Manipulation

Vision and multimodal sensing serve as core foundations for linking environmental perception to action selection in robotic manipulation. Object pose, the spatial relationship between the end-effector and the target object, depth information, and occlusion conditions directly influence manipulation success. Accordingly, robotic system evaluation should account not only for task success but also for sensing conditions, calibration, repeatability, and system fidelity. Studies on collaborative-robot vision-system evaluation and multi-sensor mobile-robot validation illustrate this system-level evaluation perspective [15,16]. Vision-based robust manipulation further links RGB-D sensing to object loss, grasp failure, contact state, and recovery decisions, indicating that sensing affects not only recognition accuracy but also failure handling in manipulation systems [17].

ROEP extends this perspective to the evaluation of VLA manipulation policies. Rather than evaluating the measurement accuracy of individual cameras or depth sensors, this study analyzes what evidence is provided by a closed-loop policy that couples image observations, language instructions, and action generation across different execution conditions. Thus, ROEP focuses on policy-level reliability and claim-level interpretation, rather than sensor-level accuracy.

### 2.2. Vision–Language–Action and Language-Conditioned Robot Policies

Language-conditioned robot policies link natural-language instructions to robot actions, enabling robots to perform diverse tasks without task-specific low-level programs. CALVIN and LIBERO provide benchmark suites that integrate language instructions, visual observations, and long-horizon manipulation, thereby establishing a foundation for language-conditioned manipulation research [12,18]. This research direction has subsequently expanded toward VLA policies and generalist robot policies. RT-2 introduced a paradigm for transferring knowledge from vision-language models to robotic action generation, while Open X-Embodiment advanced large-scale robot learning by integrating diverse robot embodiments and demonstrations [2,3]. Octo further indicates that reusing open-source generalist robot policies across heterogeneous observation spaces, action spaces, and robot platforms requires specifying not only the model itself but also the downstream runtime interfaces and fine-tuning conditions [6].

OpenVLA, X-VLA, and VLA-Adapter are the VLA models most directly examined in this study. OpenVLA provides an open-source VLA model to support reproducible research, X-VLA emphasizes cross-embodiment adaptation, and VLA-Adapter focuses on lightweight policy/bridge designs for connecting vision-language representations to action spaces in small-backbone VLA systems [4,7,8]. Whereas these studies primarily contribute in the form of model architectures, training data, adaptation strategies, or open-source implementations, ROEP provides an evaluation protocol for interpreting the results of existing VLA policies from a deployment-oriented perspective rather than proposing a new VLA model.

### 2.3. Robot Manipulation Benchmarks and Evaluation Protocols

Robot manipulation benchmarks enable different policies to be compared under shared task conditions. RLBench provides diverse robot learning tasks with visual and proprioceptive observations, while CALVIN offers an environment for evaluating language-conditioned long-horizon manipulation [18,19]. LIBERO constructs language-conditioned manipulation task suites from the perspective of knowledge transfer and lifelong robot learning; the Goal and Object tasks considered in this study are evaluated within this benchmark family [12]. Although these benchmarks are essential for policy comparison, benchmark success rate alone does not establish deployment-relevant reliability.

Deployment-oriented evaluation is inherently connected to the broader sim-to-real gap in robotic manipulation, since simulation results alone are insufficient to account for physical transferability, hardware-induced latency, calibration drift, actuator variability, and realistic sensor-noise characteristics. In this regard, ROEP should be regarded as a simulation-bounded evaluation protocol intended to clarify benchmark claims prior to physical robot validation, rather than as a substitute for empirical studies conducted under real deployment conditions.

Recent manipulation-benchmark research has moved beyond nominal success-rate comparison toward more explicit treatment of generalization, perturbations, and reproducibility. FMB evaluates generalizable robotic learning using reproducible hardware/software setups and functional manipulation tasks, whereas THE COLOSSEUM evaluates the generalization of manipulation policies across environmental perturbation axes, including object variation, texture, lighting, distractors, physical properties, and camera pose [9,20]. Benchmarking studies of VLA models across robotic learning tasks further show that action space, task complexity, and environmental factors influence how model performance should be interpreted [21]. This line of work motivates ROEP’s focus on perturbation conditions, runtime fidelity, and failure semantics rather than a single success-rate metric.

The closest neighboring research direction to ROEP is not the development of new models, but the design of evaluation protocols and benchmarking frameworks as research contributions in their own right. For example, work on LLM-based robot navigation jointly evaluates instruction parsing, inference latency, path quality, and task success, while work on intelligent medical radar proposes a protocol for systematizing evaluation targets, experimental conditions, reference measurements, and metrics [13,14]. These studies indicate that protocol design can constitute an independent contribution and that system-level evaluation depends critically on what is measured and under which conditions.

### 2.4. Robustness, Repeatability, and Deployment-Oriented Evaluation

Robustness evaluation assesses how stably a policy operates outside nominal conditions. For VLA manipulation policies, however, a success-rate drop under perturbations does not by itself explain deployment risk. Such failures must be disentangled according to whether they arise from sensitivity to visual perturbations, natural variability across repeated executions under the same condition, or runtime-interface mismatch involving the checkpoint, preprocessing pipeline, action scaling, or control mode.

Recent VLA robustness studies show that high nominal benchmark scores may not guarantee practical reliability. LIBERO-Plus analyzes the vulnerability of VLA models across perturbation dimensions including object layout, camera viewpoint, robot initial state, language instruction, lighting, background texture, and sensor noise, while LIBERO-X proposes a benchmark direction for more systematic robustness and generalization evaluation on LIBERO-family tasks [10,11]. LIBERO-PRO further introduces perturbations along manipulated objects, initial states, task instructions, and environments, showing that high scores under standard LIBERO settings may not adequately capture robust policy behavior under controlled distribution shifts [22]. These studies share the motivation behind ROEP’s perturbation testing, but ROEP differs in that it converts existing benchmark rollouts into deployment-facing claims rather than proposing a new task suite or robustness benchmark.

Another related direction concerns failure detection and recovery. SAFE proposes multitask failure detection using internal VLA features, and FailSafe connects VLA failure reasoning to executable recovery actions [23,24]. These studies indicate that post-failure judgment and recovery constitute distinct problems in VLA deployment. Rather than training a new detector or recovery model, ROEP separates rollout success from recovery/shield support and reports this distinction through protocol claim statuses, such as “Recovery-facing claim supported ” and “Recovery-facing claim withheld.”

Repeatability and reproducibility have also long been central concerns in robotics evaluation. MIRRER organizes robot experiments in terms of repeated, reproduced, and replicated evaluations, while benchmarking protocols for robot end-effectors emphasize the importance of explicitly defining metrics such as grasp strength, cycle time, and repeatability under fixed measurement conditions [25,26]. ROEP applies this repeatability perspective to VLA rollout interpretation through noise-floor control, where perturbation effects are interpreted relative to variability observed across repeated executions.

ROEP addresses these issues by adding a deployment-oriented interpretation procedure on top of existing benchmark results. It first verifies whether execution under clean conditions is reliable, interprets perturbation results together with a repeated-run noise floor, and separately reports runtime fidelity and recovery/shield support. Thus, ROEP is neither a new model competing with OpenVLA, X-VLA, or VLA-Adapter, nor a new task suite like RLBench, CALVIN, or LIBERO. Rather, it is an evaluation protocol that converts raw rollout outcomes from existing VLA policies and manipulation benchmarks into stricter and more reproducible claim-level decisions.

## 3. Materials and Methods

### 3.1. ROEP Protocol Overview

ROEP is an evaluation protocol designed to avoid summarizing a model-task pair with a single success rate. Instead, it evaluates each row through a sequence of evidence stages and progressively constrains the final deployment-facing claim. The protocol takes as input the model, checkpoint, task suite, task instruction, observation/action interface, runtime bridge, and perturbation configuration. For each rollout, ROEP records not only task success but also timeout, unsafe attempt, recovery activity, pair integrity, and runtime provenance. The final decision gate integrates these pieces of evidence and assigns one of four claim statuses: Recovery-facing claim supported, Recovery-facing claim withheld, Clean prerequisite not met, or Insufficient evidence.

In this paper, a deployment-facing claim means an interpretation that a trained VLA policy remains usable when the evaluation bridge, sensing stream, and task execution condition deviate from the exact nominal clean setting used to report success rate. It does not certify open-world deployment safety; rather, it specifies whether the reported benchmark evidence is sufficient for a robustness or recovery-facing claim under the evaluated perturbation and runtime conditions. The claim is therefore limited to the controlled LIBERO simulation setting and to the perturbation/runtime conditions explicitly evaluated in this study.

The basic unit of ROEP is a model-suite-task row. Each row is first evaluated under clean conditions. The protocol proceeds to perturbation, noise-floor, and recovery/shield support interpretation only when the clean evidence is sufficient to support downstream claim interpretation. This ordering is essential. Interpreting a row without valid basic execution under clean conditions as a perturbation-robustness failure may conflate the model’s actual weakness with runtime mismatch or checkpoint mismatch. ROEP avoids this conflation by not converting raw rollout outcomes directly into leaderboard-style scores. Instead, it sequentially applies admission, perturbation, repeatability, fidelity, and decision gates.

### 3.2. Evaluation Tasks, Policies, and Runtime Setup

The evaluation suite used in this study consists of four LIBERO Goal tasks and four LIBERO Object tasks. We evaluate OpenVLA, X-VLA, and VLA-Adapter for each suite-task combination, yielding 24 model-suite-task rows. Each clean row is evaluated over 50 episodes, and the main table reports clean SR together with n/50 support. Medium-perturbation SR and perturbation timeout-termination rate are reported for rows that are admitted after the clean-execution gate or are otherwise evaluable. In the 24-row evaluation considered in this study, no row fails clean admission and no row remains unresolved. Nevertheless, the ROEP protocol is designed to withhold downstream perturbation claims and report them as N/A when clean admission fails.

**Figure A1:**
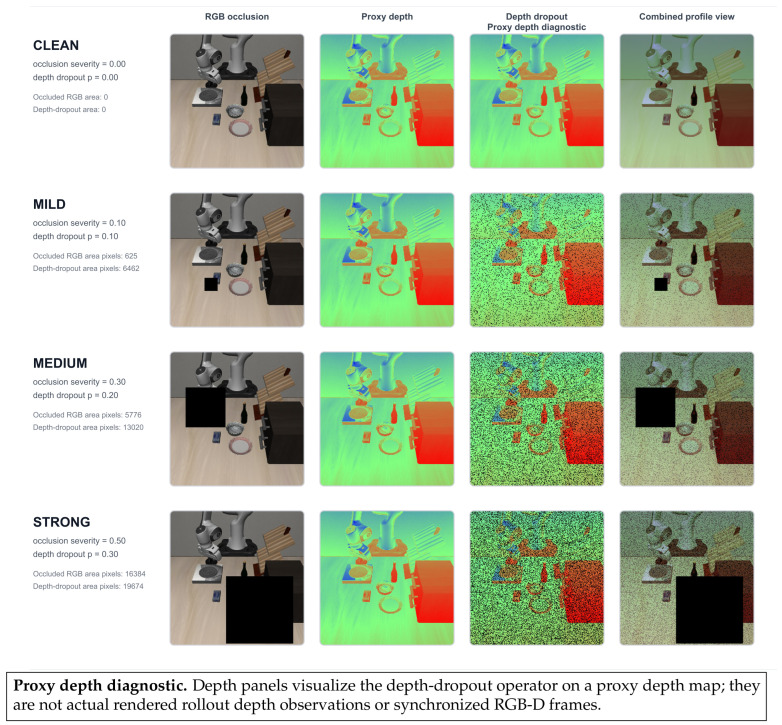
Diagnostic visualization of the perturbation profiles used in ROEP. The figure illustrates how the clean, mild, medium, and strong profiles change RGB occlusion severity and depth-channel dropout probability. Depth panels are proxy diagnostic renderings of the depth-dropout operator rather than actual rendered rollout depth observations. The combined view illustrates RGB occlusion and depth dropout as separate operator components and should not be read as a single synchronized RGB-D observation. The affected-area annotations describe the approximate diagnostic rendering footprint of the RGB and depth operators and are not performance metrics.

All perturbation results in the 24-row main table correspond to the medium profile. Accordingly, the main manuscript claim is restricted to clean-to-perturbation comparison under the medium-perturbation profile. In this context, perturbation denotes an observation-level perturbation condition compared with clean rollouts, rather than a claim of a new OOD benchmark. The mild and strong profiles were additionally evaluated with 3 models × 8 tasks × 2 profiles × 50 episodes of support, and noise-floor clones were generated with the same support. This severity sweep is used only as secondary diagnostic evidence to examine how success rate and timeout-termination rate change as perturbation severity increases; it does not alter the protocol claim status assigned under the medium condition. Figure A1 is a diagnostic figure that characterizes the severity space of the perturbation operator and should not be interpreted as a rollout-performance figure.

**Figure 1:**
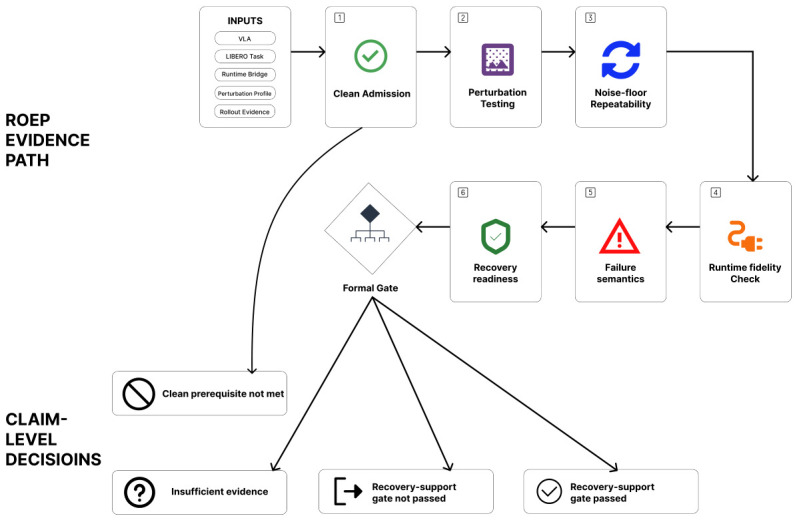
Overview of the ROEP evaluation protocol. ROEP receives a VLA policy, checkpoint/runtime configuration, task suite, observation/action interface, and perturbation profile as inputs, then routes each model-task pair through clean admission, perturbation testing, noise-floor repeatability control, runtime-fidelity checking, recovery/shield support assessment, and formal claim gating. The numbers 1–6 in the figure indicate the sequential order of these evidence-path stages. The output is a claim-status table that separates supported recovery-facing evidence from withheld or insufficient evidence.

Figure 1 summarizes the overall ROEP evidence path.

The evaluation suite consists of four LIBERO Goal tasks and four LIBERO Object tasks. The Goal tasks cover goal-conditioned object placement and environment interaction, whereas the Object tasks focus on object-specific pick-and-place behaviors. We focus on Goal and Object rather than the full set of LIBERO suites because the purpose of this study is not to maximize task-suite coverage, but to compare how the same claim protocol behaves across multiple VLA policies under controlled clean, perturbation, repeatability, runtime-fidelity, and recovery/shield support evidence. The tasks used in the evaluation are listed in Table 1.

The three models are selected because they provide public VLA policies or publicly reproducible checkpoint/runtime configurations that can be evaluated on LIBERO Goal and Object tasks while linking clean execution, perturbation testing, noise-floor estimation, and recovery/shield support within a unified ROEP evaluation record. For OpenVLA Object, we use an object-tuned LIBERO-Object checkpoint to eliminate checkpoint mismatch as a confounding factor. X-VLA uses the public X-VLA LIBERO checkpoint together with its intended runtime interface, including absolute control mode and lerobot_native image preprocessing. VLA-Adapter uses suite-matched VLA-Adapter Pro checkpoints, and its full 50-episode evaluation clean, perturbation, noise-floor, shield-bootstrap, recovery/full, and formal-gate results are integrated into the ROEP evaluation record. Runtime settings required for interpreting the results are described in Materials and Methods, and compact row-level runtime/provenance status is summarized in Appendix A.2.

### 3.3. Clean Capability Admission and Runtime Fidelity

Clean admission is the first gate in ROEP. The purpose of this stage is to check whether a model-task pair has enough nominal capability to support downstream perturbation or recovery claims. Rows failing the clean stage are withheld from perturbation-failure interpretation. Low clean success may reflect a real model limitation, but it may also result from runtime-fidelity issues such as checkpoint mismatch, preprocessing mismatch, or action-convention mismatch.

Operationally, clean admission is a minimum capability and integrity gate, not a high-performance threshold. In this study, the planned clean rollout must be executed under the intended protocol, and episode-level records and condition-level summaries must be generated without missing data. These records include episode success, termination reason, step count, and action traces. ROEP then applies a control/action integrity check to determine whether the recorded rollout contains valid policy actions. This check verifies that clean step-level records exist, the action-mode profile is recorded, numeric action traces are available, and the maximum absolute action magnitude observed over the run exceeds the nonzero tolerance $\left(10^{-6}\right)$. If this condition is not satisfied, low success is not interpreted as model capability; it is treated as insufficient runtime/control evidence.

The $10^{-6}$ tolerance serves as a numerical sanity threshold rather than a task-performance hyperparameter, distinguishing exact-zero padding, zero-action fallback, or action-logging failure. Exact-zero padding refers to a case in which the entire action vector remains zero during dimensional alignment, rather than only unused action dimensions being padded. Zero-action fallback refers to a case in which inference or action conversion fails and the runtime returns a zero-vector action to continue the rollout. Action-logging failure refers to a case in which the action actually executed or converted is not preserved as a numeric vector in step-level records, making it appear as all-zero or missing during post hoc analysis. In all three cases, the log may show $\max_{t}{∥a_{t}∥}_{\infty }$ as zero or at the level of numerical noise. ROEP therefore uses the $10^{-6}$ threshold to separate zero-like action traces. This value is much smaller than a meaningful control command on the normalized action scale, so it is not intended to penalize policies that output small actions. ROEP uses this threshold only to check whether a real numeric policy action is recorded in the rollout, not whether the policy performs well.

This check should be regarded as a minimal sanity check on the action trace and is not intended to certify full behavioral validity or to exclude all forms of stale, clipped, low-variance, or semantically incorrect nonzero action behavior. For instance, a repeated stale action or an action clipped to a numerically valid range may still satisfy this nonzero criterion; such cases should therefore be interpreted in light of runtime provenance, rollout behavior, and downstream evidence, rather than on the basis of the $10^{-6}$ threshold alone.

After record completeness and action integrity are verified, ROEP applies a minimum capability floor. In this study, clean admission requires at least one clean success and ${\mathrm{SR}}^{\left(\mathrm{clean}\right)}\geq 0.05$. The 50-episode budget is used as a fixed per-row support budget to keep model-task-profile comparisons balanced across the 24-row main evaluation, rather than as a guarantee of precise rare-event estimation. It was chosen as a practical comparison budget that balances row-level resolution and full model-task coverage. Smaller budgets would make individual episode outcomes dominate SR and HR estimates, whereas substantially larger budgets would improve uncertainty estimates but would trade off against completing the full 3-model × 8-task protocol with clean, perturbation, clone, and recovery/shield branches. With 50 clean episodes, this floor corresponds to at least 3/50 successes, so rows with only 1/50 or 2/50 successes do not open downstream perturbation or recovery claims. This criterion is a pragmatic protocol convention for minimum evaluability, not a statistical certification that a low-success row has stable capability. A row exactly at the floor would still have substantial binomial uncertainty, and ROEP therefore does not use the clean-admission floor by itself to support robustness or recovery-facing conclusions. In the final 24-row comparison, no row lies near this floor: the minimum clean SR is 0.30 (15/50), and all rows would remain clean-admitted under more conservative floors of 0.10 or 0.20. Thus, the substantive conclusions are not driven by borderline 3/50 clean-support cases. The threshold functions as an operational guardrail for opening downstream claims, not as a selector of high-performing policies. Even after clean admission, a row is not assigned the Recovery-facing claim supported status until perturbation, noise-floor, failure-semantics, shield-availability, and formal-gate checks are also satisfied.

The four binary checks used in clean admission are defined before the indicator is evaluated. $C_{\mathrm{src}}$ verifies that the planned clean rollout source exists; $C_{\log}$ verifies complete episode-, step-, and summary-level execution records; $C_{\mathrm{act}}$ verifies numeric nonzero action traces; and $C_{\mathrm{fid}}$ verifies the absence of unresolved checkpoint, preprocessing, control-mode, or action-convention mismatch. Their product equals one only when all four checks pass. The row-level clean-admission indicator for model *m* and task *k* can then be written as Equation (1):

(1) $$A_{m,k}^{\mathrm{clean}}=\left\{\begin{array}{cc} 1, & C_{\mathrm{src}}C_{\log}C_{\mathrm{act}}C_{\mathrm{fid}}=1\;\wedge \;\mathrm{success}\_{\mathrm{total}}_{m,k}^{\mathrm{clean}}\geq 1\;\wedge \;{\mathrm{SR}}_{m,k}^{\left(\mathrm{clean}\right)}\geq 0.05,\\ 0, & \mathrm{otherwise}. \end{array}\right.$$

If any integrity check fails or the minimum clean-support floor is not met, $A_{m,k}^{\mathrm{clean}}=0$, and the row is withheld as Clean prerequisite not met rather than interpreted as a perturbation or recovery failure.

Thus, ROEP preserves clean-admission failure as Clean prerequisite not met and withholds downstream perturbation or recovery claims. This design keeps the failure visible while preventing its cause from being converted into an incorrect claim. Clean admission is not a leaderboard score; it is a prerequisite for downstream claims.

Runtime-fidelity checking is a prerequisite for result interpretation in ROEP. VLA policies are sensitive to checkpoint selection, image preprocessing, prompt formatting, action scaling, action clipping, absolute/relative control mode, and end-effector orientation convention. If these elements differ from the intended runtime, low clean success may reflect an evaluation-bridge mismatch rather than a true model limitation.

In this study, X-VLA is evaluated with its intended runtime interface, using absolute control and lerobot_native image preprocessing. Under this configuration, X-VLA achieves high clean SR across Goal and Object tasks, indicating that runtime-interface fidelity is a prerequisite for interpreting VLA rollout results. OpenVLA Object is evaluated with a suite-matched object-tuned checkpoint, controlling checkpoint provenance as a potential confounding factor. VLA-Adapter is evaluated with suite-matched VLA-Adapter Pro checkpoints and the intended inference contract. The reported VLA-Adapter rows are restricted to executions that pass finite/nonzero action-integrity screening under the intended runtime path. Accordingly, the main quantitative results are interpreted only for rows that pass runtime-fidelity screening, not for arbitrary success-rate rows. Compact provenance and runtime-integrity status are summarized in Appendix A.2.

### 3.4. Perturbation Profiles and Noise-Floor Repeatability

Perturbation testing evaluates how observation-level perturbations affect policy outcomes for clean-admitted rows. Imitation-learning policies and robot policies can accumulate compounding errors that make recovery progressively harder when they encounter observations or states outside the training distribution during rollout. Thus, perturbation can be interpreted not only as visual corruption but also as an evaluation condition that pushes the policy into unfamiliar execution states [27,28]. This stage records termination semantics in addition to comparing medium-perturbation SR with clean SR. It also records which termination semantics are observed under perturbation. The same SR drop can have different deployment implications depending on whether it is associated with step-budget timeout or an unsafe-action spike. In this LIBERO evaluation, a rollout terminates when the task succeeds or when the maximum budget of 280 control steps is reached. If task success does not occur within 280 steps, the episode is recorded as a timeout.

In the 24-row medium-profile comparison reported in the main text, all perturbation non-success episodes terminate by step-budget timeout. ROEP retains logging channels for unsafe attempt, invalid execution, and safe refusal; in this LIBERO visual-perturbation setting, these channels remain secondary to step-budget timeout as sources of non-success episodes. Because no adversarial safety trigger or environment-level object-drop/collision terminal predicate is used as a primary outcome, rollouts that fail to satisfy the task-success predicate usually continue until the step budget is exhausted. The perturb timeout-termination rate reported in the main text can therefore appear numerically redundant with $1-{\mathrm{SR}}^{\left(\mathrm{perturb}\right)}$, but its role is to make the observed terminal semantics of non-success explicit.

ROEP defines perturbation intensity through a profile parameter $\lambda =(\alpha_{\mathrm{rgb}},p_{\mathrm{depth}})$, where $\alpha_{\mathrm{rgb}}$ is RGB occlusion severity and $p_{\mathrm{depth}}$ is depth dropout probability. The profile parameters used in this study are clean=(0.0,0.0), mild=(0.10,0.10), medium=(0.30,0.20), and strong=(0.50,0.30). The 24-row evaluation prioritizes model-task coverage and quantitative-comparison consistency, so it uses the medium profile as the primary perturbation condition. Therefore, medium-perturbation SR and perturb timeout-termination rate reported in the main text correspond to λ=(0.30,0.20).

RGB occlusion severity $\alpha_{\mathrm{rgb}}$ controls the relative side length of a square occlusion patch with respect to the shorter side of the image. For example, $\alpha_{\mathrm{rgb}}=0.30$ applies a black patch whose side length is approximately 30% of the shorter image dimension at a randomly sampled RGB-frame location. In the LIBERO runtime, the deterministic random state is derived from the run id, seed, episode index, and step index, so the occlusion location and depth-dropout mask are generated at the control-step level rather than as a single fixed overlay for an entire run. Depth dropout probability $p_{\mathrm{depth}}$ controls a pixel-wise dropout mask in the depth channel, and selected depth pixels are set to zero. Clean, perturbation, clone, and recovery comparisons retain the same episode support and deterministic pairing key.

Observation perturbation can be written as an operator defined by the profile parameter $\lambda =(\alpha_{\mathrm{rgb}},p_{\mathrm{depth}})$. Let $x_{t}^{\mathrm{rgb}}$ be the RGB observation, $x_{t}^{\mathrm{depth}}$ the depth observation, and $q_{t}$ the remaining proprioceptive state. Under perturbation, the observation provided to the policy is given by Equation (2):

(2) $${\tilde{o}}_{t}^{\left(\lambda \right)}=P_{\lambda }\left(o_{t}\right)=\left(O_{\alpha_{\mathrm{rgb}}}\left(x_{t}^{\mathrm{rgb}}\right),\;M_{t}^{\left(p_{\mathrm{depth}}\right)}⊙x_{t}^{\mathrm{depth}},\;q_{t}\right),\;a_{t}=\pi_{m}\;\left({\tilde{o}}_{t}^{\left(\lambda \right)},\ell_{k}\right).$$

Here, $O_{\alpha_{\mathrm{rgb}}}$ is the RGB occlusion operator, $M_{t}^{\left(p_{\mathrm{depth}}\right)}$ is the depth mask generated with dropout probability $p_{\mathrm{depth}}$, ⊙ denotes element-wise multiplication, and $\ell_{k}$ is the task language instruction. Thus, $M_{t}^{\left(p_{\mathrm{depth}}\right)}⊙x_{t}^{\mathrm{depth}}$ is the depth observation after applying the pixel-wise dropout mask. The action definition is included because the recorded action trace is reused in action-integrity and recovery/shield support analyses. The clean condition is the special case λ=(0,0), and the main comparison uses the medium profile λ=(0.30,0.20).

The perturbation modality itself is visualized in Appendix A.1 Figure A1. This diagnostic figure shows the form of the RGB occlusion and depth-channel dropout operators under clean, mild, medium, and strong profiles. It serves as an operator diagnostic rather than a rollout-outcome or model-performance figure. In the medium perturbation, RGB occlusion and depth dropout are applied together, but depth dropout is not directly visible in the RGB frame. Therefore, Appendix A.1 Figure A1 separately visualizes the depth-channel dropout operator using a proxy depth map. Mild/strong rollout outcomes are separated from this operator diagnostic and are not used as primary quantitative claims.

Because perturbation non-success in this study is observed as timeout termination rather than unsafe attempt, perturb timeout-termination rate is included in the main table. This value serves to make the terminal semantics explicit, rather than to introduce an additional failure category. It shows directly that non-success in the main condition is primarily task-completion timeout. Thus, perturbation testing is used as evidence combined with termination semantics, not as a standalone robustness score.

Noise-floor control estimates residual variability when the same condition is repeatedly executed. In robotics benchmarking, repeated execution, reproducibility, replicability, and metric repeatability are important determinants of the credibility of evaluation claims [25,26]. Even in simulation-based evaluation, results may not repeat exactly due to action decoding, controller numerical precision, simulator step timing, timeout boundaries, and GPU inference kernels. Therefore, before claiming a perturbation or recovery effect, one should first estimate how much variation can occur when the same condition is rerun.

ROEP estimates this floor using nominal clones and perturbation clones, and checks whether the final claim exceeds simple repeat-run variability. Without this procedure, a small perturbation difference can be mistaken for a real perturbation effect. Noise-floor control is a core mechanism by which ROEP produces conservative claims.

The clone run is a repeated execution of the same model-task-profile condition rather than a copied trajectory. For each model-task row, the nominal clone keeps the same policy, checkpoint/runtime configuration, task language instruction, episode index set, and clean profile as the nominal run. The perturbation clone keeps the same policy, checkpoint/runtime configuration, task language instruction, episode index set, and perturbation profile as the perturbation run, while using a separate clone run identifier. At the condition-summary level, each model-task-profile row therefore has one original summary and one clone summary for the nominal condition, and one original summary and one clone summary for the perturbation condition. Because the perturbation random state is derived from the run id, seed group, episode index, and step index, the perturbation clone provides a repeat execution under the same severity profile rather than reusing an identical pixel mask sequence. In this study, each original or clone condition is summarized from five seed groups with ten episodes per seed group, yielding 50 episodes per condition. Episode manifests preserve a deterministic pairing key, and clone-based differences are interpreted only after confirming that the original and clone supports are paired by this key. Task-level $F_{M}$ values are computed first; when *M* is success rate, the same quantity is reported as $F_{\mathrm{SR}}$ in the model-suite-level figures and tables. Model-suite summaries are then computed as arithmetic means over the four task-level values within each LIBERO suite rather than by pooling episodes across tasks.

For a metric *M*, such as success rate or timeout rate, the repeat-run floor for condition *c* can be summarized by the difference between the original run and the clone run. ROEP uses the larger of the floors observed in the nominal clone and the perturbation clone as a conservative reference. Equation (3) defines this reference; nom denotes the clean/nominal run and pert denotes the perturbation run.

(3) $$\begin{array}{cc} \Delta_{M}^{\mathrm{nom}}\; & =\left|M^{\left(\mathrm{nom}\right)}-M^{\left(\mathrm{nom}\;\mathrm{clone}\right)}\right|,\\ \Delta_{M}^{\mathrm{pert}}\; & =\left|M^{\left(\mathrm{pert}\right)}-M^{\left(\mathrm{pert}\;\mathrm{clone}\right)}\right|,\\ F_{M}\; & =\max\{\Delta_{M}^{\mathrm{nom}},\Delta_{M}^{\mathrm{pert}}\},\\ E_{M}\; & =\left|M^{\left(\mathrm{nom}\right)}-M^{\left(\mathrm{pert}\right)}\right|. \end{array}$$

Here, $\Delta_{M}^{\mathrm{nom}}$ and $\Delta_{M}^{\mathrm{pert}}$ are the repeat-run differences between the original and clone runs in the nominal and perturbation conditions, respectively. $F_{M}$ is a conservative reference value for metric variation caused by repeat execution alone, and $E_{M}$ is the observed effect size between nominal and perturbation. If $E_{M}$ is difficult to distinguish from $F_{M}$, or if paired support is low, ROEP does not promote the difference to a strong perturbation or recovery effect.

Noise-floor interpretation is not closed by a single formal hypothesis test. ROEP checks paired episode support, SR and timeout differences in baseline-clone and perturb-clone comparisons, HR decomposition, and limiting factor reasons together. If a perturbation effect is difficult to separate from clone-to-clone variability, or if paired support is low, ROEP does not produce a strong recovery or robustness claim and reports Insufficient evidence or Recovery-facing claim withheld. Thus, the noise floor is not a penalty applied to performance. It is a conservative repeatability reference that constrains whether an observed perturbation difference can be converted into a claim.

Compact summaries of repeat-run support and noise-floor interpretation are provided in Appendix A.3.

### 3.5. Metrics and Failure Semantics

ROEP retains success rate but does not use it as the sole headline metric. Let $s_{i}\in \left\{0,1\right\}$ be the task-success indicator for episode *i*. The success rate under condition *c* is defined in Equation (4):

(4) $${\mathrm{SR}}^{\left(c\right)}=\frac{1}{N_{c}}\sum_{i=1}^{N_{c}}s_{i}^{\left(c\right)}.$$

In this paper, *c* mainly denotes the clean condition and the medium-perturbation condition. Clean success rate, ${\mathrm{SR}}^{\left(\mathrm{clean}\right)}$, is used as admission evidence for downstream claims, while medium-perturbation success rate, ${\mathrm{SR}}^{\left(\mathrm{perturb}\right)}$, is used to interpret perturbation response for clean-admitted rows. Success drop under perturbation is defined in Equation (5):

(5) $$\Delta \mathrm{SR}={\mathrm{SR}}^{\left(\mathrm{clean}\right)}-{\mathrm{SR}}^{\left(\mathrm{perturb}\right)}.$$

Timeout is recorded separately. Let $\tau_{i}\in \left\{0,1\right\}$ indicate whether episode *i* terminates as a timeout without succeeding within the step budget. The timeout rate is defined in Equation (6):

(6) $${\mathrm{TimeoutRate}}^{\left(c\right)}=\frac{1}{N_{c}}\sum_{i=1}^{N_{c}}\tau_{i}^{\left(c\right)}.$$

In general, timeout rate differs from simple failure rate because unsafe attempt and timeout have different deployment implications. In the 24-row medium-profile comparison of this study, however, all perturbation non-success episodes terminate by timeout, so the perturb timeout-termination rate reported in the main outcome table (Section 4) is numerically equal to $1-{\mathrm{SR}}^{\left(\mathrm{perturb}\right)}$. We make this redundancy explicit. The main result is not that multiple terminal failure types are frequent, but that task-completion failure under medium visual perturbation terminates by step-budget timeout. Therefore, the main table focuses on clean SR, medium-perturbation SR, clean-to-perturb ΔSR, perturb timeout-termination rate, protocol claim status, and primary limiting factor. HR decomposition and provenance summaries are reported in Appendix Table A2 and Table A4, together with the model-suite aggregate results presented later in the Results.

ROEP also computes harm between paired conditions. If an episode that succeeded under a baseline condition *b* fails under a comparison condition *c*, it is treated as counterfactual harm. Harm rate is defined in Equation (7):

(7) $${\mathrm{HR}}^{\left(c|b\right)}=\frac{\sum_{i}1\left[s_{i}^{\left(b\right)}=1\wedge s_{i}^{\left(c\right)}=0\right]}{\sum_{i}1\left[s_{i}^{\left(b\right)}=1\right]}.$$

HR can be decomposed by failure type. For example, timeout-induced harm is defined in Equation (8):

(8) $${\mathrm{HR}}_{\mathrm{timeout}}^{\left(c|b\right)}=\frac{\sum_{i}1\left[s_{i}^{\left(b\right)}=1\wedge \tau_{i}^{\left(c\right)}=1\right]}{\sum_{i}1\left[s_{i}^{\left(b\right)}=1\right]}.$$

The same form defines ${\mathrm{HR}}_{\mathrm{unsafe}}$. In the 24-row evaluation, ${\mathrm{HR}}_{\mathrm{unsafe}}$ is observed as zero. This metric is therefore used not as evidence that unsafe-action harm was found, but as an audit channel confirming that the observed harm decomposes into the timeout channel rather than the unsafe-attempt channel. Unsafe-related gates are retained because ROEP is a deployment-oriented protocol rather than a scoring rule fitted only to the present experiment. Other tasks, perturbation families, or models may produce unsafe attempts, so the channel is kept in the gate. In the present LIBERO visual-perturbation setting, however, unsafe-related harm is not observed, and the dominant non-success pattern is step-budget timeout termination rather than unsafe-action escalation. For readability, the main table prioritizes protocol claim status, core support columns, and primary limiting factors, while representative task-level HR decomposition is reported in Table A4 and model-suite-level aggregate support is reported in the Results (Section 4).

Recovery/shield activity is recorded not as stable recovery success itself, but as support evidence for how often the shield or recovery layer intervenes during rollout. Let $z_{i}\in \left\{0,1\right\}$ indicate whether at least one shield/recovery action is applied in episode *i*. The intervention episode rate is defined in Equation (9):

(9) $${\mathrm{InterventionRate}}^{\left(c\right)}=\frac{1}{N_{c}}\sum_{i=1}^{N_{c}}z_{i}^{\left(c\right)}.$$

Let $u_{i}$ be the number of steps in episode *i* where a shield/recovery action is actually applied. Mean intervention steps are defined in Equation (10):

(10) $${\mathrm{MeanInterventionSteps}}^{\left(c\right)}=\frac{1}{N_{c}}\sum_{i=1}^{N_{c}}u_{i}^{\left(c\right)}.$$

In the execution records, recovery_rate corresponds to InterventionRate, and mean_recovery_action_applied_steps corresponds to MeanInterventionSteps. These field names are retained for traceability to the execution artifacts, but the manuscript interprets them as recovery/shield activity support rather than recovery success rates. The recovery/shield analysis in this study does not define recovery completion as a separate terminal event; instead, it records whether the shield or recovery layer actually intervenes during rollout. Thus, intervention episode rate and mean intervention steps are support metrics for the recovery-facing branch, not stable-recovery outcome metrics.

The distinction between metrics included in the main table and metrics used as supporting evidence is summarized in Appendix A.1.

### 3.6. Recovery/Shield Support and Claim-Level Decision Rule

In ROEP, recovery-facing claims and the shield module are related but not identical. A recovery-facing claim is the manuscript-level statement that a row provides enough evidence for the recovery/full evaluation branch. The shield module is used here as a risk/intervention support mechanism for that branch: a lightweight risk predictor followed by a projection/refusal operator. We use recovery/shield support as the umbrella term for the evidence needed to interpret this branch, including shield-checkpoint availability, risk-positive support, calibration, intervention activity, and auditable runtime records. The shield architecture itself is not the primary contribution of this paper, and the reported shield outputs are not recovery success rates, safety-certification metrics, or stand-alone detector-performance results. The shield equations and output traces are included to make the recovery/shield support evidence reproducible, not to claim a new safety controller. The evaluation contribution is that ROEP reports rollout performance separately from recovery/shield support and checks whether a model-task row provides enough evidence to train, calibrate, activate, and audit a recovery-facing branch before assigning a recovery-facing claim status. This distinction is needed because high task success can coexist with insufficient evidence for the shield branch, and therefore may not support a recovery-facing claim.

For rows in which recovery/full evaluation is attempted, the system connects a shield module corresponding to the model-task-condition. The shield module has two parts. First, it uses a lightweight risk predictor that takes the low-dimensional observation vector recorded at each time step and the action proposed by the policy, and outputs a risk score. Second, if the risk score is greater than the calibration threshold τ, a projection/refusal layer conservatively modifies the action, or returns a safe-refusal action if the modified action is still judged unsafe. The shield does not replace the policy. If the nominal action remains below the threshold, the original action passes through unchanged.

Mathematically, let $o_{t}^{\mathrm{low}}$ be the low-dimensional observation at time step *t*, $a_{t}$ the action proposed by the policy, $I_{t}^{\mathrm{shield}}$ the intervention indicator, and ${\tilde{a}}_{t}$ the action passed to execution by the shield. The shield module is summarized in Equation (11):

(11) $$\begin{array}{cc} r_{t} & =\sigma \;\left(f_{\theta }\;\left(\mathrm{zscore}\;\left([o_{t}^{\mathrm{low}};a_{t}]\right)\right)\right),\;I_{t}^{\mathrm{shield}}=1\left[r_{t}\geq \tau \right],\\ {\tilde{a}}_{t} & =\left\{\begin{array}{cc} a_{t}, & I_{t}^{\mathrm{shield}}=0,\\ \Pi_{\mathrm{safe}}\left(a_{t};r_{t}\right), & I_{t}^{\mathrm{shield}}=1. \end{array}\right. \end{array}$$

Here, $r_{t}$ is the shield risk score, $f_{\theta }$ is the lightweight risk predictor, zscore(·) denotes normalization using training statistics, τ is the calibration threshold, $I_{t}^{\mathrm{shield}}$ indicates whether the shield intervenes, and $\Pi_{\mathrm{safe}}$ is the projection/refusal operator. Thus, the shield does not generate a new policy action; it intervenes only when the policy action exceeds the risk threshold.

The threshold τ is calibrated once for each task-specific shield and is held fixed during a rollout; only the step-level risk score $r_{t}$ changes over time.

Shield training uses step-level execution records generated from rollouts. Features are formed by concatenating the low-dimensional observation and proposed action at each step. Labels are derived from episode outcomes. Steps from successful episodes are used as the safe class, while episodes in which unsafe attempt is observed are used as a primary source of the risk class. When unsafe-attempt support is insufficient, timeout episodes are used as a fallback risk source. In risk episodes, a short tail window immediately before terminal failure can be treated as risk steps, while the remaining prefix can be used as weak safe support. This is because failure is not uniformly distributed over the episode; it is often expressed more directly in the action sequence near the terminal phase. Because unsafe-attempt events are not observed in the main 24-row visual-perturbation evaluation, the fallback labels used here should be interpreted as terminal-risk support derived mainly from timeout-tail behavior, not as evidence of a broadly calibrated unsafe-action detector. Consequently, the Recovery-facing claim supported status should not be read as validation of the shield predictor itself; it means that the available recovery/shield support satisfies the reporting rule used by ROEP for the evaluated condition.

Training and calibration are performed using episode-level splits. If steps from the same episode are mixed simultaneously into train, validation, and calibration sets, step-level leakage can occur. Therefore, the split is fixed by episode identifier, using an approximately 0.8/0.1/0.1 train/validation/calibration partition keyed by episode identifier. The risk predictor is trained as a lightweight multilayer perceptron with z-score normalization. In the implementation used for the reported VLA-Adapter shield bootstrap, the MLP has hidden layers with dimensions 128,64,32, ReLU activations after each hidden linear layer, and a final scalar logit converted to a risk score by the sigmoid function in Equation (11). Training uses Adam with learning rate $10^{-3}$, batch size 256, a maximum of 40 epochs, and early stopping on validation loss with patience 7. The training metadata preserves the effective label sources, train/validation/calibration sample counts, class counts, completed epochs, learning rate, batch size, and hidden dimensions for each task-specific shield. Let $x_{j}=\mathrm{zscore}\left(\left[o_{j}^{\mathrm{low}};a_{j}\right]\right)$ be the step-level training sample, $y_{j}\in \left\{0,1\right\}$ the risk label, and $r_{j}=\sigma \left(f_{\theta }\left(x_{j}\right)\right)$ the predictor output. Equation (12) gives the weighted binary cross-entropy objective used to compensate for class imbalance:

(12) $$\min_{\theta }\;L_{\mathrm{shield}}\left(\theta \right)=\frac{1}{|D_{\mathrm{train}}|}\sum_{j\in D_{\mathrm{train}}}w_{y_{j}}\left[-y_{j}\log r_{j}-\left(1-y_{j}\right)\log\left(1-r_{j}\right)\right].$$

Here, $y_{j}=1$ denotes a risk step, $y_{j}=0$ denotes a safe step, and $w_{y_{j}}$ is the class-specific weight for correcting class imbalance. The calibration threshold τ is set to the empirical upper quantile of safe calibration scores as shown in Equation (13):

(13) $$\tau =Q_{1-\alpha }\left(\{\;r_{j}:j\in D_{\mathrm{cal}},\;y_{j}=0\;\}\right).$$

The VLA-Adapter shield bootstrap uses a risk-tail horizon of 5 steps and an α=0.05 calibration setting. The task-specific shield checkpoint and calibration threshold τ are preserved for each supported task.

**Figure A2:**
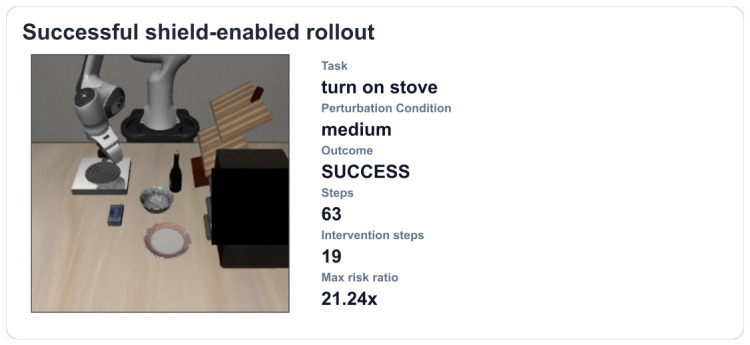
Representative shield-output trace, rollout view. The frame and summary values come from a full-medium VLA-Adapter stove episode selected to illustrate recorded shield-intervention activity. This panel identifies the rollout context used for the shield-output trace in Figure A3 and Figure A4.

**Figure A3:**
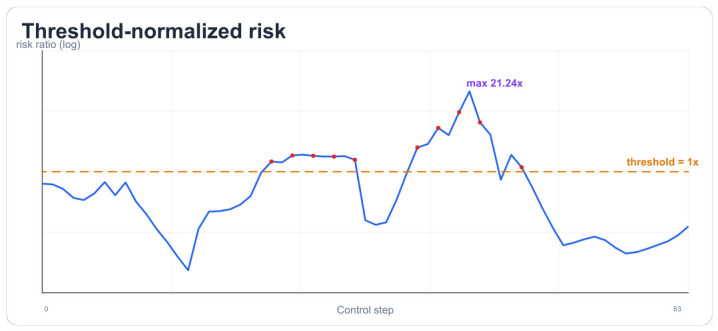
Representative shield-output trace, risk-score view. The curve plots the threshold-normalized risk score $r_{t}/\tau$; the horizontal reference line marks $r_{t}/\tau =1$, red markers denote steps where the shield intervened, and the peak label reports $\max_{t}r_{t}/\tau$. Raw $r_{t}$ values are task-specific calibration scores, not calibrated risk probabilities.

**Figure A4:**
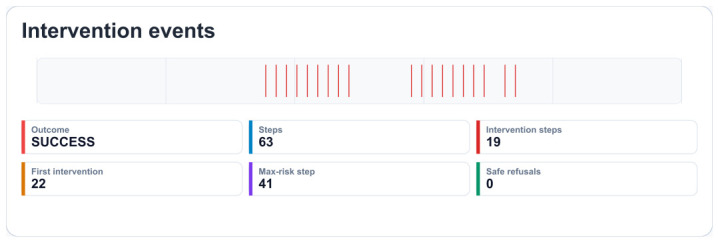
Representative shield-output trace, intervention-event view. The vertical ticks show the same intervention steps marked by red points in Figure A3. The event summary reports the rollout outcome, episode length, intervention count, first intervention step, max-risk step, and safe-refusal count.

At runtime, the shield computes the risk score at every step. If the risk score is lower than τ, the action is not modified. If the risk score is greater than or equal to τ, the projection layer conservatively reduces the translational action and slightly raises the end-effector, then checks action bounds and maximum translation norm again. If the action remains non-finite after projection or the risk is not sufficiently reduced, the shield may return a safe-refusal action. At each control step, ROEP records the shield-output tuple $\left(r_{t},\tau ,I_{t}^{\mathrm{shield}},{\mathrm{reason}}_{t},{\mathrm{proj}}_{t}\right)$, where ${\mathrm{reason}}_{t}$ stores the intervention reason and ${\mathrm{proj}}_{t}$ stores projection metadata. At the rollout level, these step-level outputs are summarized as intervention rate and mean intervention steps. Therefore, whether the shield intervened, and on what support a recovery/full claim was made, can be traced after the rollout. A representative shield-output trace is provided in Appendix A.1 Figure A2, Figure A3 and Figure A4, and aggregate intervention activity is summarized in Table A5.

In this manuscript, shield functioning is assessed operationally rather than as stand-alone detector accuracy. A shield is considered operationally functioning when the task-specific checkpoint is available, the calibration threshold τ is loaded, the step-level risk score $r_{t}$ is computed during rollout, threshold crossings are recorded as $I_{t}^{\mathrm{shield}}=1$, and the intervention reason and projection metadata are preserved in the rollout logs. Table A5 summarizes these checks at the task level, and Figure A2, Figure A3 and Figure A4 show the corresponding single-rollout trace. This operational check is distinct from validating the shield as a general unsafe-action detector.

Shield unavailability withholds the recovery-facing claim while leaving rollout capability assessment intact. Shield training requires support from both safe and risk classes. If risk-positive support is insufficient for a given task, shield bootstrap may fail even when the underlying rollout capability is strong. In this case, ROEP does not reduce the row to model failure; instead, it marks the recovery claim as withheld. In the VLA-Adapter evaluation, task-specific shield checkpoints and τ values were generated for seven of the eight tasks, while put_the_bowl_on_the_plate did not produce a shield because risk-positive support was insufficient. Thus, the claim-withheld decision for that row is interpreted as a recovery/shield support limitation, not as absence of rollout capability.

Shield bootstrap status, checkpoint availability, τ, intervention count, and recovery activity are used as support evidence for the protocol claim status in the main text. Compact task-level summaries are placed in Appendix A.3.

The formal gate integrates clean admission, perturbation outcome, noise-floor evidence, runtime-fidelity evidence, failure semantics, and recovery/shield support. The reported protocol claim statuses are Recovery-facing claim supported, Recovery-facing claim withheld, Clean prerequisite not met, and Insufficient evidence. Recovery-facing claim supported is a manuscript-level evidence-sufficiency status: it means that the row has passed the predefined checks required to report a recovery-facing protocol claim under the evaluated LIBERO condition. It is not a recommendation to deploy the policy, not a certification that the shield is generally safe or effective, and not a recovery-success result. Recovery-facing claim withheld means that the clean prerequisite is satisfied, but a predefined formal-gate condition fails, or shield-checkpoint/risk-positive support is insufficient for a recovery-facing claim. Clean prerequisite not met means that the nominal-capability or runtime-fidelity prerequisite is not satisfied, so downstream claims are withheld. Insufficient evidence means that observed evidence support is too low or paired support is insufficient for a strong conclusion.

Intuitively, the four statuses answer what kind of manuscript claim can be made, not whether the policy should be deployed. A row with high clean and perturbation SR can still be Recovery-facing claim withheld if recovery/shield support is unavailable or the formal gate is not satisfied. A row that fails the clean prerequisite stops before perturbation failure is interpreted. A row marked Insufficient evidence is not declared a model failure; it indicates that the available paired or observed support is too limited for a strong recovery-facing claim.

The decision precedence is fixed explicitly. If clean admission fails, the row does not proceed to the formal gate and is terminated as Clean prerequisite not met. For rows that pass clean admission, any pair mismatch or insufficient clean/medium paired-support denominator leads to Insufficient evidence. If shield checkpoint, risk-positive support, or recovery/shield activity evidence does not support the branch-level claim, the row is marked as Recovery-facing claim withheld. The gate then checks paired harm, unsafe harm, full-vs-perturb SR drop, and recovery-activity criteria; only rows satisfying all required evidence checks are marked as Recovery-facing claim supported. Thus, Recovery-facing claim supported is a reporting authorization for the evaluated protocol claim, not a performance grade, deployment action, or shield validation result.

The numerical gate criteria in this study are not universal safety-certification thresholds, nor are they tuned model-ranking criteria. They are conservative reporting rules fixed before manuscript-level interpretation for a 50-episode paired LIBERO evaluation. Their purpose is to prevent recovery-facing claims from being made from isolated successes, nearly empty paired support, or a recovery/full branch that worsens the perturbation-condition outcome. The clean SR floor prevents one- or two-success rows from opening downstream perturbation or recovery-facing claims. The paired-denominator requirement prevents formal decisions from being made from unstable paired comparisons. Because HR estimates are discrete paired proportions, rows near the minimum denominator remain uncertain: at the denominator floor of five paired-support episodes, one episode changes HR by 0.20. Therefore, the denominator rule is a minimum reporting guardrail rather than a precision guarantee, and rows near or below the floor are interpreted as support-limited. The full-vs-perturb SR-drop bound prevents the recovery/full branch from being treated as claim-supporting when it substantially reduces success relative to the perturb-only condition. Therefore, the gate criteria define when ROEP is allowed to report a recovery-facing protocol claim under the evaluated condition, rather than certifying general safety, deployment readiness, or recovery success.

Specifically, the gate requires ${\mathrm{HR}}_{\mathrm{total}}\leq 0.08$ and ${\mathrm{HR}}_{\mathrm{unsafe}}\leq 0.03$ for the clean recovery-vs-baseline paired comparison. Under the medium condition, the full-vs-perturb comparison checks that the unsafe-attempt rate does not increase relative to perturb-only execution, that full SR drops by no more than 0.10 relative to perturb SR, and that the noise-floor-adjusted ${\mathrm{HR}}_{\mathrm{unsafe}}\leq 0.30$. Pair mismatch must be zero for both clean and medium paired comparisons, and the clean and medium HR denominators must each provide at least 5 paired-support episodes. For branches that require recovery/shield activity to support a claim, the full-medium mean shield-intervention steps must be at least 0.1, and shield-checkpoint availability is interpreted together with risk-positive support. These values are operational guardrails for conservative claim reporting under finite 50-episode support, not universal constants; transferring them to other task suites, episode budgets, or physical-robot settings will require task- and platform-specific calibration. Detailed decision rules and thresholds are summarized in Table A1; primary limiting factors are included in the final column of the main outcome table presented in the Results (Section 4), while compact support and decomposition values are summarized in Table A4 and Table A5.

Table 2 summarizes these reported protocol claim statuses together with their implementation trace labels and operational meanings.

### 3.7. Implementation and Reproducibility

The ROEP implementation stores each rollout as separate episode-level records, step-level records, run-level summaries, and episode manifests. Episode-level records include task identifier, instruction, perturbation profile, seed, success status, termination reason, and step count. Step-level records include action traces, control/action mode, observation-perturbation metadata, and safety or recovery signals. Run-level summaries report episode count, success count, success rate, timeout-related aggregate values, unsafe indicators, and perturbation parameters. Episode manifests preserve deterministic pairing keys so that clean, perturbation, noise-floor, and recovery comparisons can be interpreted over the same episode support.

The ROEP evaluation record in this study produces machine-readable tables together with completeness metadata. The record contains 24 model-suite-task rows, and no row used in the main outcome table has unresolved verification. The main paper includes the full 24-row outcome table and compact support needed for claim interpretation. This structure makes ROEP results auditable protocol outputs rather than a single leaderboard table.

## 4. Results

### 4.1. Evaluation Overview

The Results section separates two kinds of outcomes. Some observations are protocol-by-design consequences of ROEP, such as the fact that high clean or medium-perturbation SR does not automatically authorize a recovery-facing protocol claim when paired support, shield availability, or formal-gate evidence is insufficient. Other observations are empirical findings specific to the evaluated models, tasks, runtime interfaces, and LIBERO perturbation conditions, such as the timeout-dominant VLA-Adapter Object-suite degradation. We report both types, but interpret them differently: protocol-by-design outcomes validate the intended behavior of the evidence gate, whereas empirical findings characterize the evaluated policies under the specified sensor-to-action runtime and perturbation conditions.

**Figure 2:**
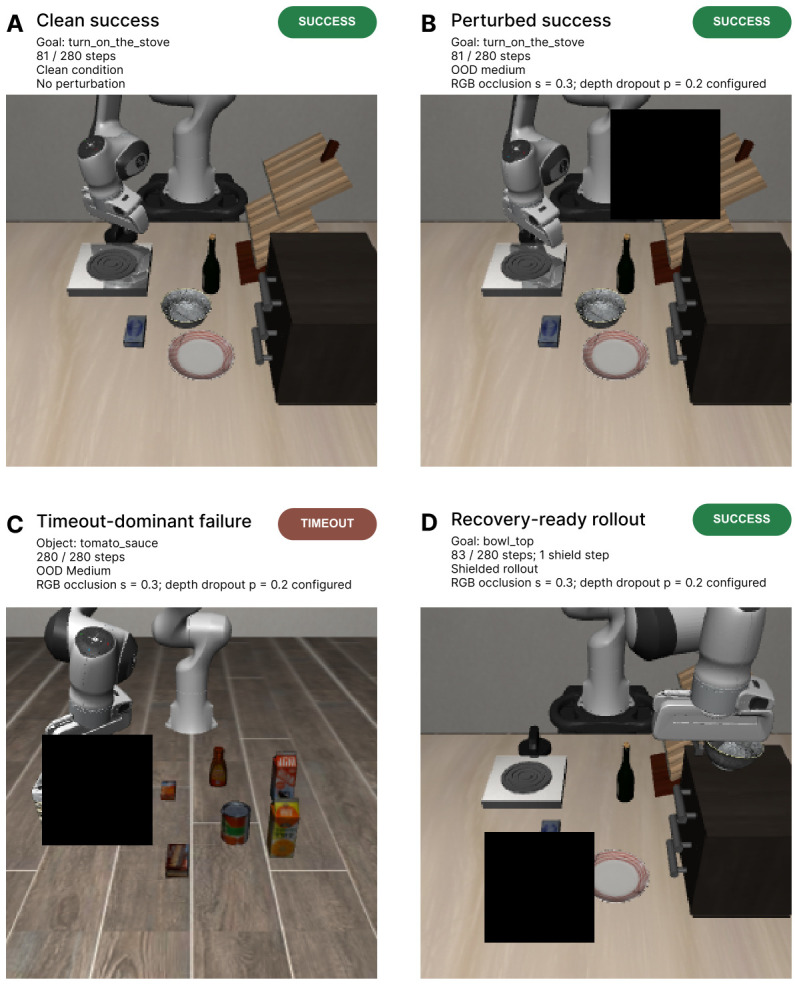
Representative rollout evidence used to interpret ROEP outcomes. (Panel **A**) shows a clean successful rollout, (Panel **B**) shows a successful rollout under the medium-perturbation profile, (Panel **C**) shows a perturbation-induced timeout case, and (Panel **D**) shows a shield-enabled rollout with recorded intervention support. The figure is intended as qualitative evidence for the execution conditions summarized quantitatively in Table 3.

**Figure 3:**
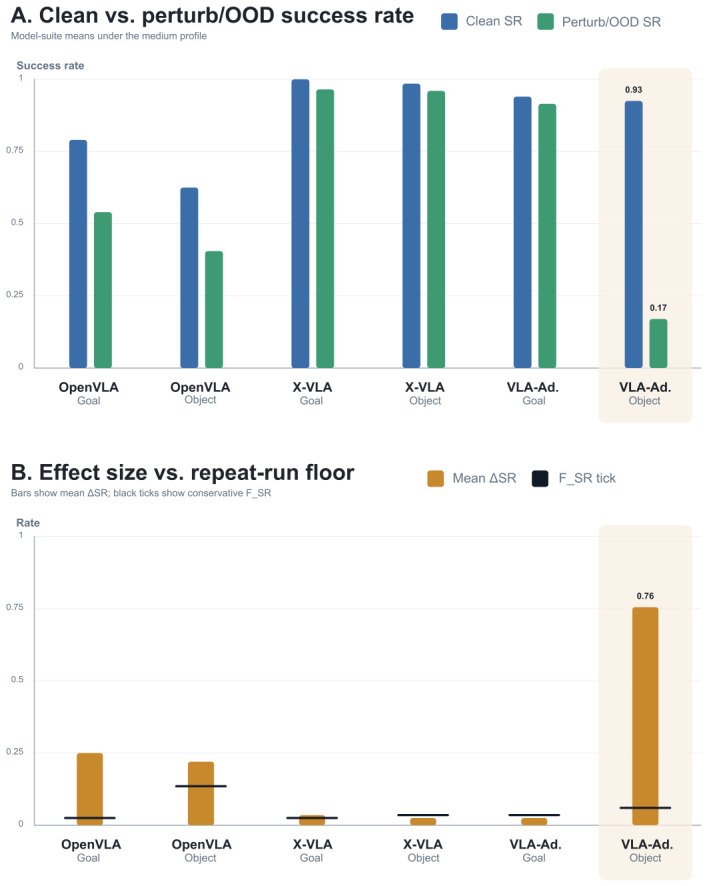
Aggregate clean-to-perturbation effect relative to the repeat-run floor. Panel (**A**) compares mean clean and medium-perturbation success rates by model and suite. Panel (**B**) compares $\Delta \mathrm{SR}={\mathrm{SR}}^{\left(\mathrm{clean}\right)}-{\mathrm{SR}}^{\left(\mathrm{medium}\right)}$ against $F_{\mathrm{SR}}$, where $F_{\mathrm{SR}}$ is a conservative repeat-run reference estimated from clone–original variability. When ΔSR exceeds $F_{\mathrm{SR}}$, ROEP treats the perturbation effect as more distinguishable from finite-run repeatability; when it is comparable to or smaller than $F_{\mathrm{SR}}$, the difference is interpreted conservatively as potentially within repeat-run variability. (**A**) Clean and medium-perturbation success rates by model and suite. (**B**) Clean-to-perturbation effect size relative to the repeat-run floor. ΔSR is clean SR minus medium-perturbation SR, and $F_{\mathrm{SR}}$ is the conservative repeat-run reference.

**Figure 4:**
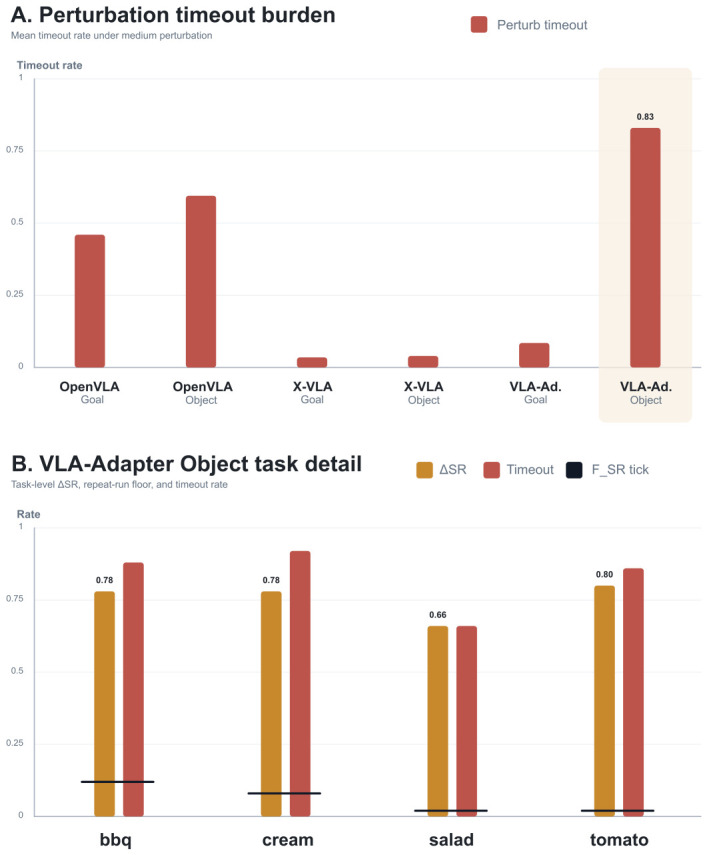
Timeout-dominant failure semantics under medium perturbation. Panel (**A**) summarizes timeout-termination rates by model and suite. Panel (**B**) focuses on VLA-Adapter Object tasks by jointly displaying ΔSR, $F_{\mathrm{SR}}$, and timeout-termination rate, showing that clean success remains high while the medium-perturbation drop exceeds the repeat-run reference and non-success is dominated by timeout termination. (**A**) Medium-perturbation timeout-termination rate by model and suite. (**B**) VLA-Adapter Object-suite task-level detail comparing ΔSR, $F_{\mathrm{SR}}$, and timeout-termination rate.

The results are organized to keep these layers visible. Table 3 provides the primary row-level outcome matrix, Figure 2 gives representative rollout evidence, Figure 3 and Figure 4 summarize perturbation and timeout patterns, and Table 4 aggregates the model-suite-level support.

The 24-row ROEP evaluation in this study consists of OpenVLA, X-VLA, and VLA-Adapter evaluated on four LIBERO Goal tasks and four LIBERO Object tasks. The main outcome table has no unresolved verification rows, and no row is missing clean SR or medium-perturbation SR. Of the 24 rows, 16 receive the Recovery-facing claim supported status, 7 receive the Recovery-facing claim withheld status, and 1 is marked as Insufficient evidence. No row in the main outcome table is Clean prerequisite not met. Runtime/checkpoint provenance is summarized separately in Appendix A.2.

These results show that ROEP provides more granular protocol claim statuses than a simple ranking. Rows with the same success rate can differ in whether they support or withhold a recovery-facing protocol claim. Therefore, protocol claim status is not another name for success rate and does not prescribe a deployment action; it is a protocol-level reporting status that passes through multiple evidence stages.

The main interpretations of Table 3 are threefold. First, under intended runtime handling, X-VLA shows high clean SR and medium-perturbation SR in most rows and small ΔSR, but some rows remain Recovery-facing claim withheld because of formal-gate or shield-support issues. Second, with suite-matched checkpoint provenance, OpenVLA provides interpretable Object-suite results, but some tasks still show substantial ΔSR and perturbation timeout-termination rates. Third, VLA-Adapter provides additional evidence from a third evaluated model on the Goal suite, but the Object suite shows large ΔSR and perturbation timeout termination after clean admission. Thus, Table 3 is less a model-ranking table than an evidence table that shows how ROEP separates rollout performance from deployment-facing claims.

In Table 3, the VLA-Adapter Goal rows for goal_bowl_top and goal_stove show ΔSR=−0.02 and −0.04, respectively. These values correspond to one or two additional successes under the medium-perturbation profile relative to the clean run under 50-episode support. Such small negative ΔSR values appear only in near-ceiling rows. Without a mechanistic analysis of occlusion placement, visual attention, and trajectory differences, they should not be interpreted as evidence of perturbation-induced improvement. The $F_{\mathrm{SR}}$ reference is used as a conservative repeat-run floor for suppressing overinterpretation, not as a causal explanation for why the perturbation run obtained additional successes.

Figure 2 provides representative qualitative rollout evidence for the same execution conditions summarized in Table 3. The purpose of this figure is not to add another quantitative metric, but to show what the clean, medium-perturbation, timeout, and shield-enabled execution modes look like at the rollout level. This visual evidence helps connect the numerical outcome matrix to concrete observation/action traces.

### 4.2. Clean Admission and Runtime-Fidelity Outcomes

X-VLA illustrates the role of runtime-interface fidelity as an evaluation condition. Under the intended adapter/runtime configuration, including absolute control and lerobot_native image preprocessing, X-VLA achieves a mean clean SR of 0.992 across all eight rows. All four Goal tasks achieve clean SR 1.00, and the Object suite achieves mean clean SR 0.985. These results support treating checkpoint, preprocessing, control mode, and action convention as part of the evaluated sensor-to-action system rather than incidental implementation details.

OpenVLA Object is evaluated with the LIBERO-Object-suite-matched checkpoint, which makes the Object-suite rows interpretable under controlled checkpoint-provenance assumptions. The clean SRs for OpenVLA Object are 0.70 for bbq_sauce, 0.30 for cream_cheese, 0.60 for salad_dressing, and 0.90 for tomato_sauce, and three of the four rows receive the Recovery-facing claim supported status.

VLA-Adapter passed clean admission for all eight tasks in the full 50-episode evaluation. Its overall mean clean SR is 0.933, with mean clean SR 0.940 on the Goal suite and 0.925 on the Object suite. The rows reported in the manuscript are restricted to the intended FP32 runtime path and pass finite, nonzero, unique action-integrity screening. This separates runtime-integrity screening from the success-rate and protocol-claim interpretation reported in Table 3.

### 4.3. Perturbation and Timeout-Termination Pattern

Perturbation outcomes differ substantially across models. X-VLA shows the most stable perturbation response under its intended runtime interface, with mean medium-perturbation SR 0.963 and mean perturb timeout-termination rate 0.037 across the eight rows. OpenVLA has mean clean SR 0.708, mean medium-perturbation SR 0.473, and mean perturb timeout-termination rate 0.527, showing relatively high timeout termination under perturbation. VLA-Adapter has a strong overall mean clean SR of 0.933, but mean medium-perturbation SR decreases to 0.542 and mean perturb timeout-termination rate increases to 0.458.

The VLA-Adapter result differs sharply by suite. On the Goal suite, it shows strong perturbation response, with mean medium-perturbation SR 0.915 and mean perturb timeout-termination rate 0.085. In contrast, on the Object suite, mean clean SR remains high at 0.925, but mean medium-perturbation SR drops to 0.170 and mean perturb timeout-termination rate rises to 0.830. Therefore, the VLA-Adapter Object result should be interpreted not as absence of clean capability, but as a case where task completion stability is strongly reduced into timeout under the perturbation condition.

The Object-suite drop of VLA-Adapter is more pronounced than that of the other two VLA policies. X-VLA Object remains almost stable, moving from mean clean SR 0.985 to medium-perturbation SR 0.960, with perturb timeout-termination rate 0.040. OpenVLA Object drops from mean clean SR 0.625 to medium-perturbation SR 0.405, but this case includes rows with low clean capability by task. In contrast, VLA-Adapter Object has strong nominal execution with mean clean SR 0.925, yet medium-perturbation SR sharply falls to 0.170. Thus, this result should be interpreted not as low baseline performance continuing under perturbation, but as a policy that can execute cleanly yet loses completion stability under the Object-task medium-perturbation condition.

This difference suggests task-family-dependent sensitivity under the evaluated observation-degradation operator. LIBERO Object tasks have an object-centric manipulation structure that requires identifying a specific object instance, grasping it, and moving it to the target location. The RGB occlusion and depth-channel dropout of the medium-perturbation profile can partially remove visual cues such as the target object, receptacle, or grasp geometry. In this study, VLA-Adapter Object non-success appears as timeout termination rather than unsafe-action spikes. Therefore, it is more appropriate to interpret the perturbation as destabilizing task progress and completion timing rather than immediately stopping the policy. This interpretation does not claim a general architectural limitation of VLA-Adapter; it presents suite-conditioned perturbation sensitivity observed under the ROEP protocol. The VLA-Adapter Object-suite drop should therefore be read as an empirical sensitivity observed under the suite-matched VLA-Adapter Pro checkpoint and intended FP32 runtime path, rather than as a checkpoint-independent property of the model family. Although action-integrity and runtime-fidelity checks reduce the likelihood that the result is a simple execution artifact, they do not rule out checkpoint- or interface-dependent sensitivity. Additional checkpoints and runtime variants would be needed to separate model-family robustness from configuration-specific effects.

This result shows why ROEP requires failure semantics. It is more deployment-relevant to distinguish whether low medium-perturbation SR is associated with unsafe-attempt increase or step-budget timeout than to state only that the success rate is low. In the 24-row medium comparison, all perturbation non-success episodes are observed as timeout terminations. Therefore, the main table includes perturb timeout-termination rate together with medium-perturbation SR to make explicit that failures are not distributed across diverse unsafe/refusal/invalid terminal events, but instead terminate as task-completion timeouts.

**Table 4:** Model-suite aggregate support for perturbation effect analysis.

Model	Suite	Mean Clean SR	Mean MediumPerturb. SR	Mean Signed ΔSR	Mean Absolute $E_{\mathrm{SR}}$	Mean $E_{\mathrm{SR}}$	Rows $|\Delta \mathrm{SR}|>F_{\mathrm{SR}}$	Mean Perturb Timeout Rate	Mean HR_timeout_	Mean HR_unsafe_
OpenVLA	Goal	0.790	0.540	0.250	0.250	0.025	4/4	0.460	0.242	0.000
	Object	0.625	0.405	0.220	0.220	0.135	3/4	0.595	0.445	0.000
X-VLA	Goal	1.000	0.965	0.035	0.035	0.025	2/4	0.035	0.088	0.000
	Object	0.985	0.960	0.025	0.025	0.035	1/4	0.040	0.167	0.000
VLA-Adapter	Goal	0.940	0.915	0.025	0.055	0.035	3/4	0.085	0.107	0.000
	Object	0.925	0.170	0.755	0.755	0.060	4/4	0.830	0.823	0.000

$F_{\mathrm{SR}}$ is the conservative repeat-run reference used to contextualize clean-to-perturbation changes. Mean HR_unsafe_=0.000 indicates that unsafe harm was not observed in this evaluation; the dominant harm channel is timeout-related, as shown by Mean HR_timeout_ and perturbation timeout-termination rates.

The noise-floor reference changes how clean-to-perturbation drops are interpreted. For X-VLA Object, the observed mean ΔSR is close to the repeat-run reference, so ROEP treats the perturbation-specific effect as weak. In contrast, the VLA-Adapter Object drop substantially exceeds $F_{\mathrm{SR}}$, supporting a cautious interpretation that the observed Object-suite drop is larger than the conservative repeat-run reference under the clone comparison. This comparison is the main reason Figure 3 and Figure 4 and Table 4 are interpreted together rather than as separate descriptive summaries. The $F_{\mathrm{SR}}$ comparison is used to qualify the interpretation of clean-to-perturbation differences; it is not a formal hypothesis test or a universal statistical significance threshold.

### 4.4. Claim Status Outcomes and Recovery/Shield Support

Protocol claim status is not identical to success rate. For example, X-VLA Object salad_dressing has clean SR 1.00, medium-perturbation SR 1.00, and perturb timeout-termination rate 0.00, but the recovery/full branch support is not sufficient, so the row is marked as Recovery-facing claim withheld. This case is a protocol-by-design outcome rather than an empirical claim that the rollout policy is weak: clean/perturb rollout performance alone does not authorize a recovery-facing claim. ROEP checks not only success rates in Table 3, but also paired harm, SR drop, unsafe-related signals, pairing integrity, shield availability, and recovery-activity support.

Recovery-facing claim withheld can arise through two routes. First, the recovery/full branch may execute but fail a predefined formal-gate threshold. Second, the recovery/full branch may not be evaluable because the shield checkpoint or risk-positive support is insufficient. Timeout termination is important failure-semantics evidence for interpreting these rows, but it is not a standalone final-status threshold. For example, VLA-Adapter Object bbq_sauce and tomato_sauce are marked as Recovery-facing claim withheld because the formal gate does not pass the clean recovery-vs-baseline ${\mathrm{HR}}_{\mathrm{total}}$ criterion. In contrast, VLA-Adapter Goal bowl_plate has high clean and perturbation SR, but its recovery claim is withheld because shield-bootstrap support is insufficient.

The zero unsafe-related signal in this evaluation makes the failure semantics more specific. The observed degradation is closer to task-completion timeout under perturbation than to an increase in unsafe attempts. For this reason, Table 3 reports perturb timeout-termination rate together with medium-perturbation SR, while Appendix A.3 Table 4 and Table A4 separate ${\mathrm{HR}}_{\mathrm{timeout}}$ from ${\mathrm{HR}}_{\mathrm{unsafe}}$.

Among the eight X-VLA rows, five receive the Recovery-facing claim supported status and three receive the Recovery-facing claim withheld status. Among the eight OpenVLA rows, seven receive Recovery-facing claim supported and one receives Recovery-facing claim withheld. Among the eight VLA-Adapter rows, four receive Recovery-facing claim supported, three receive Recovery-facing claim withheld, and one is marked as Insufficient evidence. This distribution shows that ROEP provides more granular claim states than a simple ranking.

### 4.5. Model-Specific Findings

OpenVLA receives the Recovery-facing claim supported status on all four Goal-suite tasks, and with suite-matched checkpoint provenance it receives the same status on three of the four Object-suite tasks. However, its mean Object perturbation timeout rate remains 0.595, so checkpoint provenance control makes the Object rows interpretable but does not remove completion-stability problems under perturbation.

X-VLA shows the highest clean and perturbation performance under the intended adapter/runtime interface. However, even a row such as X-VLA Object salad_dressing, where both clean SR and medium-perturbation SR are 1.00, can remain Recovery-facing claim withheld if recovery/full branch support is insufficient. Thus, the X-VLA results show both the importance of runtime-fidelity control and the distinction between rollout performance and recovery claims.

VLA-Adapter provides additional third-model evidence on the Goal suite, but on the Object suite it shows a large increase in perturbation timeout-termination rate after clean admission. The bbq_sauce, cream_cheese, and tomato_sauce tasks all pass clean admission, but under medium perturbation their timeout rates rise to 0.86–0.92, and their protocol claim statuses are Recovery-facing claim withheld or Insufficient evidence. Therefore, VLA-Adapter strengthens model coverage while serving as a key case showing how ROEP separates timeout-termination patterns.

### 4.6. Secondary Perturbation-Severity Analysis

The main result of the manuscript is fixed to the 24-row medium-perturbation profile comparison, but an additional secondary sweep was run to examine perturbation-severity response. This sweep uses OpenVLA, X-VLA, and VLA-Adapter on the eight LIBERO Goal/Object tasks, with 50-episode support for each profile, and completes all 48 rows. There are no missing, failed, or unresolved verification rows, and clone-based noise-floor tables were generated with the same support. This result serves as secondary diagnostic evidence showing the direction of severity response relative to the medium-based main claim, while Table 3 remains the primary outcome matrix.

A compact numerical summary of the severity sweep is provided in Table A6. At the model level, all three models maintain some success under the mild profile, but success rates decrease strongly under the strong profile: OpenVLA 0.068, X-VLA 0.320, and VLA-Adapter 0.237. At the suite level, the strong profile is most severe for the Object suite. OpenVLA Object and VLA-Adapter Object drop to zero success across all four tasks under the strong profile, while X-VLA Object retains a nonzero success rate of 0.125. In contrast, X-VLA and VLA-Adapter retain partial residual performance on the Goal suite under strong perturbation.

Noise-floor clone results are used as a conservative caveat for interpreting severity response. Pair support is 50 and pair mismatch is 0 for all 48 rows. The mean absolute clone-original SR deltas are 0.060 for OpenVLA mild, 0.005 for OpenVLA strong, 0.008 for X-VLA mild, 0.060 for X-VLA strong, 0.032 for VLA-Adapter mild, and 0.042 for VLA-Adapter strong. The mild-to-strong drop is larger than the average clone-original delta, so the direction of severity response is visible. However, small task-level differences should still be interpreted conservatively together with clone deltas.

## 5. Discussion

The Discussion follows the same distinction used to frame the Results. Some findings are consequences of ROEP’s protocol design: clean and perturbation success alone do not authorize a recovery-facing claim when paired support, shield availability, or formal-gate evidence is insufficient. Other findings are empirical observations from the evaluated policies and conditions, such as the VLA-Adapter Object-suite timeout increase under the medium RGB-D degradation profile. The discussion therefore interprets protocol-by-design outcomes as checks on the claim-reporting structure, and empirical outcomes as contingent evidence about the evaluated model, checkpoint/runtime configuration, task suite, and perturbation condition.

### 5.1. Success Rate Is Necessary but Not Sufficient

Success rate remains necessary evidence in ROEP. Clean SR is important admission evidence for opening downstream claims. However, the evaluation shows that success rate alone cannot determine a deployment-facing claim. A row such as X-VLA Object salad_dressing, where both clean and perturbation SR are 1.00, can still remain Recovery-facing claim withheld if recovery/full branch support is insufficient. Conversely, for a row with low clean SR, one must first separate nominal-capability or runtime-fidelity issues before discussing perturbation failure.

ROEP retains success rate and repositions it together with admission, perturbation response, noise floor, failure semantics, and recovery/shield support. This structure avoids overstated deployment claims while not undervaluing model performance.

### 5.2. Runtime Fidelity Changes the Interpretation of Failures

One of the most important lessons from this evaluation is that runtime fidelity changes failure interpretation. X-VLA achieves high clean and perturbation performance only when evaluated through the intended adapter/runtime configuration, and OpenVLA Object requires the suite-matched object-tuned checkpoint for interpretable Object-suite evidence. This means that checkpoint, preprocessing, control mode, and action convention are not merely implementation details in VLA evaluation; they are prerequisites for scientific claims.

VLA-Adapter provides the same lesson. The reported rows are restricted to executions that pass finite, nonzero action-integrity screening under the intended runtime path. ROEP first presents performance and protocol claim status in Table 3, and separates checkpoint/runtime provenance and row-level runtime-integrity status in Appendix A.2. Without this separation, true model limitations and evaluation-bridge mismatches would be mixed into the same failure category. At the same time, passing runtime-integrity screening does not make the VLA-Adapter Object-suite degradation checkpoint-independent.

### 5.3. Interpreting Withheld Recovery-Facing Claims

A row that receives the Recovery-facing claim withheld status in the main outcome table does not correspond to a single failure mode. Some rows have their recovery claim withheld because recovery/full branch paired support or threshold conditions are insufficient, while other rows cannot produce a recovery claim because a model-specific shield checkpoint is unavailable. Therefore, a Recovery-facing claim withheld row should not be interpreted simply as “the model failed”.

The purpose of ROEP is to preserve these distinctions and separately report rollout capability, perturbation fragility, and recovery/shield support availability. This distinction is particularly important for deployment evaluation. Recent work on VLA failure detection and recovery shows that detecting failure or generating recovery actions is a distinct problem from nominal task success [23,24]. In pre-deployment judgment, whether a policy can perform a task and whether recovery or safety layers can extend the claim are separate questions.

### 5.4. Sensing Degradation as a Deployment-Facing Evaluation Axis

The perturbation profiles in ROEP should be read as controlled diagnostic sensing-degradation probes within a closed-loop sensor-to-action evaluation protocol rather than as exhaustive physical sensor-failure models. RGB occlusion approximates partial visual obstruction or loss of task-relevant visual evidence, whereas depth-channel dropout approximates missing or unreliable depth measurements. These operators complement, but do not replace, physical sensor validation by providing a compact way to test whether a VLA policy’s reported success rate remains interpretable when RGB-D observations are degraded in a controlled manipulation setting. Future versions of the protocol can extend this sensing axis to illumination changes, depth-calibration drift, camera-pose offsets, camera noise, and cross-modal synchronization errors. In structured industrial or service-robot settings, such a protocol can support pre-deployment risk analysis by identifying whether failures are dominated by sensing degradation, runtime-interface mismatch, timeout termination, or insufficient recovery support.

### 5.5. Limitations

This study has several limitations. First, the evaluation focuses on LIBERO-based simulation, so it does not cover all sensor noise, actuation latency, hardware wear, and human-in-the-loop constraints of physical robot deployment. ROEP therefore does not solve physical transfer, hardware calibration, real sensor-noise modeling, or deployment-time safety validation. Its role in this manuscript is to make simulation benchmark claims more explicit and conservative before physical-robot studies are attempted. This limitation is consistent with broader neural-network-augmented control settings, where transferring a control or evaluation scheme to a concrete platform requires platform-specific assumptions, calibration, and validation [29]. The 50-episode support is used to provide a consistent protocol-level comparison across model-task rows; it is not intended to estimate rare safety-event probabilities with high precision. Second, VLA-Adapter is evaluated with full 50-episode evaluation protocol support, but in the Object suite some rows are reported as Recovery-facing claim withheld or Insufficient evidence because perturbation success support is low. Therefore, these results should be interpreted as timeout termination and support limitation under perturbation, not as absence of clean capability in VLA-Adapter. Third, stable recovery-timing or recovery-success metrics are not used for the main claims. The recovery/shield values reported in this study are support evidence for shield-intervention activity, not recovery success rates. In addition, because unsafe-attempt events are not observed in the main visual-perturbation evaluation, some shield risk labels rely on timeout-tail fallback support. The resulting shield-output traces should therefore be interpreted as auditable intervention-support evidence for ROEP’s recovery-facing gate, not as precision/recall/F1 validation of a general unsafe-action detector. Because trained shields use timeout-tail fallback labels when unsafe-attempt support is absent, the 16 rows assigned the Recovery-facing claim supported status should be interpreted as rows satisfying ROEP’s recovery/shield support rule under the available proxy-label regime. They should not be interpreted as rows for which the shield has been validated to detect unsafe attempts, guarantee successful recovery, or certify deployment safety. Fourth, for rows without recovery/shield support, rollout performance and recovery-facing support must be interpreted separately.

Fifth, the main perturbation result focuses on the medium profile. ROEP defines mild, medium, and strong profiles, and this study also completes a mild/strong severity sweep with 50-episode support. However, the primary 24-row comparison and protocol claim status are designed to compare 3 models × 8 tasks under the same medium-perturbation profile. Therefore, mild/strong results are separated as secondary diagnostic evidence rather than evidence that changes the main manuscript claim. In particular, the strong profile creates zero-success saturation in some Object tasks, so it should be interpreted as a severity stress test rather than a model ranking. Task-level differences are qualified with clone noise-floor results.

Despite these limitations, ROEP provides more conservative and reproducible deployment-facing claims than success-only evaluation. By explicitly including runtime fidelity and claim withholding in the results, ROEP allows the gap between nominal score and practical reliability, emphasized by recent VLA robustness benchmarks, to be interpreted more carefully [9,10,11].

## 6. Conclusions

This paper proposes ROEP, a deployment-oriented evaluation protocol for robotic manipulation Vision–Language–Action policies under controlled RGB-D observation degradation and runtime variation in the sensor-to-action loop. ROEP combines clean admission, perturbation testing, noise-floor repeatability control, runtime-fidelity checking, failure semantics, and formal gating to convert raw rollout outcomes into deployment-facing protocol claim statuses.

In the 24-row ROEP evaluation of OpenVLA, X-VLA, and VLA-Adapter on eight LIBERO Goal/Object tasks, ROEP distinguishes runtime-fidelity issues, timeout-termination perturbation patterns, recovery/shield support limitations, and claim-withholding outcomes that are not visible from success rate alone. In particular, intended runtime-interface alignment is essential for interpreting X-VLA results, and suite-matched checkpoint selection is essential for interpreting OpenVLA Object results. The VLA-Adapter full 50-episode evaluation provides additional evidence from a third evaluated model on the Goal suite while also showing that perturbation timeout-termination rate can increase substantially on the Object suite after clean admission.

These results show that deployment-oriented VLA evaluation should report not only success rate but also runtime fidelity, repeatability, failure semantics, and recovery/shield support. ROEP functions as a claim-clarification protocol rather than a new leaderboard, clarifying which deployment-relevant claims are supported or withheld by available evidence. Future ROEP extensions should evaluate physical-robot deployments, broader sensing-degradation families, explicit recovery-success and recovery-timing metrics, and task-dependent calibration of claim-gate thresholds.

## Figures and Tables

**Table 1:** LIBERO Goal and Object tasks used in the 24-row ROEP evaluation.

Task ID	Suite	LIBERO Instruction
goal_cream_cheese	Goal	put the cream cheese in the bowl
goal_bowl_top	Goal	put the bowl on top of the cabinet
goal_stove	Goal	turn on the stove
goal_bowl_plate	Goal	put the bowl on the plate
object_bbq_sauce	Object	pick up the bbq sauce and place it in the basket
object_cream_cheese	Object	pick up the cream cheese and place it in the basket
object_salad_dressing	Object	pick up the salad dressing and place it in the basket
object_tomato_sauce	Object	pick up the tomato sauce and place it in the basket

**Table 2:** Operational semantics of ROEP claim statuses.

Protocol Claim Status	Implementation Trace Label	Operational Meaning
Recovery-facing claim supported	recovery_ready	The row has sufficient clean, perturbation, repeatability, runtime-fidelity, and recovery/shield support to report a recovery-facing protocol claim under the evaluated LIBERO condition; this status is not a deployment recommendation, shield certification, or recovery-success result.
Recovery-facing claim withheld	recovery_blocked _with_cause	The clean prerequisite is satisfied, but a predefined formal-gate condition or shield/recovery support availability does not allow a recovery-facing claim.
Clean prerequisite not met	clean_not _admitted	The clean condition does not establish the prerequisite for downstream claims.
Insufficient evidence	inconclusive_ low_support	Observed evidence or paired-denominator support is too low for a strong claim.

Implementation trace labels are retained only for reproducibility. The protocol claim statuses are the terms used in the manuscript. Recovery-facing claim supported is an evidence-sufficiency status for reporting the evaluated recovery-facing protocol claim; it should not be interpreted as a deployment recommendation, shield certification, recovery-success result, or general deployment-safety guarantee.

**Table 3:** Main 24-row outcome matrix for the ROEP evaluation.

Model	Suite	Task ID	Clean SR	Medium Perturb. SR	ΔSR	Perturb. Timeout Rate	Protocol Claim Status	Primary Limiting Factor
OpenVLA	Goal	goal_cream_cheese	0.64 (32/50)	0.38 (19/50)	0.26	0.62	Recovery-facing claim supported	–
OpenVLA	Goal	goal_bowl_top	0.88 (44/50)	0.60 (30/50)	0.28	0.40	Recovery-facing claim supported	–
OpenVLA	Goal	goal_stove	0.98 (49/50)	0.90 (45/50)	0.08	0.10	Recovery-facing claim supported	–
OpenVLA	Goal	goal_bowl_plate	0.66 (33/50)	0.28 (14/50)	0.38	0.72	Recovery-facing claim supported	–
OpenVLA	Object	object_bbq_sauce	0.70 (35/50)	0.56 (28/50)	0.14	0.44	Recovery-facing claim withheld	Recovery/full support
OpenVLA	Object	object_cream_cheese	0.30 (15/50)	0.10 (5/50)	0.20	0.90	Recovery-facing claim supported	–
OpenVLA	Object	object_salad_dressing	0.60 (30/50)	0.56 (28/50)	0.04	0.44	Recovery-facing claim supported	–
OpenVLA	Object	object_tomato_sauce	0.90 (45/50)	0.40 (20/50)	0.50	0.60	Recovery-facing claim supported	–
X-VLA	Goal	goal_cream_cheese	1.00 (50/50)	0.92 (46/50)	0.08	0.08	Recovery-facing claim withheld	Formal gate/recovery-full support
X-VLA	Goal	goal_bowl_top	1.00 (50/50)	1.00 (50/50)	0.00	0.00	Recovery-facing claim withheld	Formal gate/recovery-full support
X-VLA	Goal	goal_stove	1.00 (50/50)	0.98 (49/50)	0.02	0.02	Recovery-facing claim supported	–
X-VLA	Goal	goal_bowl_plate	1.00 (50/50)	0.96 (48/50)	0.04	0.04	Recovery-facing claim supported	–
X-VLA	Object	object_bbq_sauce	0.96 (48/50)	0.94 (47/50)	0.02	0.06	Recovery-facing claim supported	–
X-VLA	Object	object_cream_cheese	1.00 (50/50)	0.94 (47/50)	0.06	0.06	Recovery-facing claim supported	–
X-VLA	Object	object_salad_dressing	1.00 (50/50)	1.00 (50/50)	0.00	0.00	Recovery-facing claim withheld	Recovery/full support
X-VLA	Object	object_tomato_sauce	0.98 (49/50)	0.96 (48/50)	0.02	0.04	Recovery-facing claim supported	–
VLA-Adapter	Goal	goal_cream_cheese	0.84 (42/50)	0.76 (38/50)	0.08	0.24	Recovery-facing claim supported	–
VLA-Adapter	Goal	goal_bowl_top	0.96 (48/50)	0.98 (49/50)	−0.02	0.02	Recovery-facing claim supported	–
VLA-Adapter	Goal	goal_stove	0.96 (48/50)	1.00 (50/50)	−0.04	0.00	Recovery-facing claim supported	–
VLA-Adapter	Goal	goal_bowl_plate	1.00 (50/50)	0.92 (46/50)	0.08	0.08	Recovery-facing claim withheld	Shield bootstrap support
VLA-Adapter	Object	object_bbq_sauce	0.90 (45/50)	0.12 (6/50)	0.78	0.88	Recovery-facing claim withheld	Clean recovery-vs-baseline harm
VLA-Adapter	Object	object_cream_cheese	0.86 (43/50)	0.08 (4/50)	0.78	0.92	Insufficient evidence	Medium paired-support denominator
VLA-Adapter	Object	object_salad_dressing	1.00 (50/50)	0.34 (17/50)	0.66	0.66	Recovery-facing claim supported	–
VLA-Adapter	Object	object_tomato_sauce	0.94 (47/50)	0.14 (7/50)	0.80	0.86	Recovery-facing claim withheld	Clean recovery-vs-baseline harm

Task IDs are defined in Table 1. ΔSR is defined as clean SR minus medium-perturbation SR. The protocol claim status is a recovery-facing evidence status, not a model ranking. The primary limiting factor column is reported for rows that are Recovery-facing claim withheld or Insufficient evidence.

## Data Availability

This study used publicly available LIBERO benchmark tasks, which are available through the LIBERO project website at https://libero-project.github.io/ (accessed on 23 July 2026). The evaluation results supporting the findings are reported in the article and Appendix tables. No additional public dataset was introduced in this study.

## Abbreviations

    The following abbreviations are used in this manuscript:

ROEP	Deployment-oriented evaluation protocol for robotic manipulation VLA policies
VLA	Vision–Language–Action
SR	Success rate
HR	Harm rate
OOD	Out-of-distribution
RGB-D	Red-green-blue and depth observation
EEF	End-effector
MLP	Multilayer perceptron

## Appendix A. Protocol Details and Supporting Evidence

### Appendix A.1. Perturbation Profiles, Shield-Output Trace, and Metric Usage

Figure A1 visually defines the perturbation profiles used in ROEP. The clean profile denotes the nominal observation with RGB occlusion severity and depth dropout probability both set to zero. The mild, medium, and strong profiles are set to (0.10,0.10), (0.30,0.20), and (0.50,0.30), respectively. The first value denotes RGB occlusion severity, and the second value denotes depth-channel dropout probability. The RGB panels show the visible occlusion operator on rollout observations, whereas the depth panels are proxy diagnostic renderings of the depth-dropout operator rather than actual rollout depth observations. The combined view is used to show RGB occlusion and depth dropout as separate operator components and should not be read as a single synchronized RGB-D observation. The affected-area annotation in Figure A1 is an auxiliary visual label indicating approximately how many pixels are affected by RGB occlusion or depth dropout in the diagnostic rendering; it is not a rollout-performance metric. Therefore, Figure A1 is a protocol/operator diagnostic rather than a figure for comparing model performance.

Figure A2, Figure A3 and Figure A4 define the intended visual form of the shield-output trace. Unlike Table A5, which aggregates shield activity across tasks and suites, these figures provide a single-rollout diagnostic view split across readable panels. Their purpose is to show how the step-level shield-output tuple $\left(r_{t},\tau ,I_{t}^{\mathrm{shield}},{\mathrm{reason}}_{t},{\mathrm{proj}}_{t}\right)$ can be inspected visually. Because $r_{t}$ and τ are task-specific calibration scores rather than calibrated probabilities, the risk-trace panel plots the threshold-normalized score $r_{t}/\tau$ over control steps. The horizontal reference line is therefore $r_{t}/\tau =1$. The max risk ratio is $\max_{t}r_{t}/\tau$, and red markers indicate intervention steps where $I_{t}^{\mathrm{shield}}=1$. These figures are not used as additional performance metrics; they document the practical appearance of the shield evidence defined in Materials and Methods. Accordingly, Figure A2, Figure A3 and Figure A4 and Table A5 should be read as diagnostic records of shield-output activity, not as validation of a complete safety controller or as recovery-success evidence.

The clean SR, medium-perturbation SR, ΔSR, timeout rate, HR decomposition, noise-floor reference, and shield-intervention activity defined in Section 3 serve distinct reporting roles in the manuscript. Table 3 prioritizes the readable main evidence: clean SR, medium-perturbation SR, ΔSR, perturb timeout-termination rate, protocol claim status, and primary limiting factor. Model-suite aggregate support is placed in Table 4. Representative HR decomposition and shield/recovery activity are reported in Appendix A.3. A separate metric-role table is not retained because it would duplicate the metric definitions in Materials and Methods; instead, Table A1 reports the formal gate rules that actually determine the protocol claim status.

**Table A1:** Formal gate decision-rule summary.

Stage	Check	Threshold or Rule	If Not Satisfied	Rationale
Clean prerequisite	Clean admission indicator	$A_{m,k}^{\mathrm{clean}}=1$	Clean prerequisite not met	Nominal execution and runtime integrity are prerequisites for downstream claims.
Pairing integrity	Clean and medium pair mismatch	0 mismatches	Insufficient evidence	Paired harm and noise-floor comparisons require the same episode support.
Paired support	Clean and medium HR denominator	≥5 paired-support episodes	Insufficient evidence	At the denominator floor, one paired episode changes HR by 0.20, so near-floor rows are support-limited.
Clean recovery harm	Recovery-vs-baseline HR	${\mathrm{HR}}_{\mathrm{total}}\leq 0.08$	Recovery-facing claim withheld	Recovery/full execution should not introduce excessive paired harm.
Clean unsafe harm	Recovery-vs-baseline unsafe HR	${\mathrm{HR}}_{\mathrm{unsafe}}\leq 0.03$	Recovery-facing claim withheld	The recovery branch should not introduce unsafe-attempt harm under clean support.
Medium full-vs-perturb effect	Full SR drop relative to perturb SR	≤0.10	Recovery-facing claim withheld	The full condition should not substantially reduce perturb-condition success.
Medium unsafe harm	Noise-floor-adjusted unsafe HR	≤0.30	Recovery-facing claim withheld	Unsafe-attempt harm is guarded even when observed failures are mostly timeouts.
Shield/risk support	Shield checkpoint and risk-positive support	Required when the recovery/shield branch is used	Recovery-facing claim withheld	Recovery-facing claims require a trained shield or sufficient support to justify why the branch is unavailable.
Recovery activity	Mean shield-intervention steps	≤0.1 when the branch requires shield activity	Recovery-facing claim withheld	Recovery/shield claims require evidence that the shield branch was exercised.

The thresholds are conservative reporting rules for this 50-episode paired LIBERO evaluation. They are not universal safety-certification criteria or model-ranking thresholds; they specify when ROEP permits a recovery-facing protocol claim under the evaluated condition. HR estimates with small paired denominators are discrete and high-variance; at the minimum denominator of five, a single paired episode changes HR by 0.20.

### Appendix A.2. Runtime Fidelity and Provenance Summary

This subsection defines the provenance fields used to interpret runtime fidelity. In VLA evaluation, rollout behavior depends not only on the model name but also on checkpoint selection, image preprocessing, control mode, action scaling and clipping, gripper and end-effector conventions, and the runtime bridge connecting policy outputs to the LIBERO action interface. These fields are reported to make clear which execution assumptions underlie the success-rate rows; they are not additional performance metrics.

In the 24-row evaluation, OpenVLA Object is interpreted under a suite-matched object-tuned checkpoint, X-VLA under its intended absolute control and lerobot_native preprocessing configuration, and VLA-Adapter under suite-matched VLA-Adapter Pro checkpoints with the intended FP32 runtime path and finite/nonzero action-integrity screening. This provenance status is not used as a primary evidence column in Table 3; it is supporting information for runtime-fidelity interpretation. The corresponding fields are summarized in Table A2. Thus, the zero unsafe-related signal should be interpreted as “not observed under the implemented unsafe-attempt logging channel,” not as evidence that unsafe behavior could not occur in physical deployment.

**Table A2:** Runtime fidelity and provenance fields.

Field	Meaning
Model	OpenVLA, X-VLA, or VLA-Adapter.
Suite	LIBERO Goal or LIBERO Object.
Task	Task identifier.
Checkpoint source	Model checkpoint or repository used for evaluation.
Runtime bridge	Adapter/runtime connecting policy outputs to the LIBERO action interface.
Image preprocessing	Image preprocessing mode expected by the model.
Control mode	Absolute or relative action convention.
Action convention	Action scaling, clipping, gripper, and EEF orientation convention.
Runtime/provenance status	Whether the row is interpreted under the intended checkpoint, preprocessing, control mode, action convention, runtime bridge, and action-integrity screening.

This table defines the provenance fields used to interpret runtime-fidelity and runtime-integrity status. It is not a performance table; it records which execution assumptions must be checked before success-rate rows are interpreted as model behavior.

### Appendix A.3. Recovery/Shield and Failure-Decomposition Support

The final ROEP protocol claim status is not another name for a single metric threshold. ROEP jointly considers clean admission, perturbation evidence, repeatability evidence, runtime fidelity, failure semantics, and recovery/shield availability. Therefore, rows with the same clean SR or medium-perturbation SR can receive different protocol claim statuses depending on recovery/shield support, formal-gate output, and low-support conditions. Table 2 defines the operational meaning of each protocol claim status, and Table A3 summarizes how those definitions are distributed across model-suite groups in the 24-row evaluation.

**Table A3:** Claim-status distribution by model and suite.

Model	Suite	Recovery-Facing Claim Supported	Recovery-Facing Claim Withheld	Insufficient Evidence	Clean Prerequisite Not Met
OpenVLA	Goal	4	0	0	0
	Object	3	1	0	0
X-VLA	Goal	2	2	0	0
	Object	3	1	0	0
VLA-Adapter	Goal	3	1	0	0
	Object	1	2	1	0

Counts are aggregated over the four tasks in each model-suite group. No row in the final 24-row evaluation is Clean prerequisite not met.

Table A3 summarizes the distribution of protocol claim statuses across model-suite groups. In the final 24-row evaluation, there was no “Clean prerequisite not met” row; therefore, the clean prerequisite count is 0 for all model-suite groups. OpenVLA and X-VLA are mostly categorized as either Recovery-facing claim supported or Recovery-facing claim withheld, while VLA-Adapter Object includes one Recovery-facing claim supported row, two Recovery-facing claim withheld rows, and one Insufficient evidence row. This indicates that the limitation of VLA-Adapter Object was not due to a failure of the clean prerequisite, but rather to timeout termination under medium perturbation and limitations in formal/shield support.

This subsection summarizes the HR decomposition defined in Materials and Methods together with residual variability that can arise from repeat execution alone. Before claiming a perturbation or recovery effect, ROEP checks whether failure is explained by timeout or unsafe attempt, and also checks how much change can occur when the same condition is rerun. This procedure does not assume that simulation repeats perfectly deterministically. It clarifies that the defined metrics are used as actual claim support rather than simple logging fields.

The noise-floor clone procedure used for $F_{M}$ can be summarized as follows. For each model *m*, task *k*, and profile *c*, ROEP executes the original condition using the fixed seed-group and episode-index support. It then executes one clone run per condition with the same policy, checkpoint/runtime configuration, task instruction, episode index set, and perturbation profile, but with a separate clone run identifier. The episode manifest preserves the deterministic pairing key so original and clone episodes can be compared pairwise. ROEP computes original–clone differences for the nominal and perturbation conditions and sets $F_{M}=\max\left(\Delta_{M}^{\mathrm{nom}},\Delta_{M}^{\mathrm{pert}}\right)$. When *M* is success rate, this quantity is denoted $F_{\mathrm{SR}}$ in the model-suite-level figures and tables. Model-suite summaries are computed by first calculating task-level $F_{\mathrm{SR}}$ values and then averaging the four task-level values within each LIBERO suite; episodes are not pooled across tasks for this aggregation.

Appendix Table A3, Table A4 and Table A5 divide recovery/shield and failure-decomposition support into three compact tables. Table A3 summarizes the distribution of reported protocol claim statuses by model and suite. Table A4 compares OpenVLA, X-VLA, and VLA-Adapter on the representative Object task pick_up_the_cream_cheese_and_place_it_in_the_basket using actual numerical support. This task is useful for failure decomposition because X-VLA maintains high clean/perturbation performance, while OpenVLA and VLA-Adapter show large perturb timeout-termination rates. Table A5 provides task-level shield bootstrap and intervention-support diagnostics corresponding to the single-rollout trace in Figure A2, Figure A3 and Figure A4. Primary limiting factors for Recovery-facing claim withheld or Insufficient evidence rows are included in the final column of Table 3, and model-suite aggregate support is placed in Table 4 instead of repeated as an Appendix table.

**Table A4:** Representative Object-task numerical support.

Model	Clean SR	Medium Perturb. SR	Perturb. Timeout Rate	HR Full vs. Perturb (Seed-Macro)	Aggregate Paired Support	HR_timeout_	HR_unsafe_	Nominal Clone SR Delta	Perturb Clone SR Delta
OpenVLA	0.300	0.100	0.900	0.600	5 ^†^	0.600	0.000	0.000	0.060
X-VLA	1.000	0.940	0.060	0.043	47	0.043	0.000	0.000	0.040
VLA-Adapter	0.860	0.080	0.920	0.667	4 ^†^	0.667	0.000	0.080	0.020

HR values are seed-macro means over paired comparisons. Paired support reports the aggregate denominator across seeds. ^†^ Rows with aggregate paired support below 10 are low-confidence HR estimates; they are reported for transparency but interpreted as support-limited evidence rather than stable estimates of the underlying harm probability. At the formal denominator floor of five paired-support episodes, one episode changes HR by 0.20; rows below that floor are not used for strong recovery-facing claims.

Table A4 shows how the HR-family metrics defined in Section 3 support claim-status interpretation. For all three models, ${\mathrm{HR}}_{\mathrm{unsafe}}$ is 0 and the observed harm is decomposed into timeout. VLA-Adapter in particular passes clean admission with clean SR 0.860, but under medium perturbation it drops to medium-perturbation SR 0.080 with perturb timeout-termination rate 0.920. This supports the interpretation that Object-suite fragility is closer to timeout termination than to an unsafe-action spike. HR values in Table A4 are seed-macro means over seed-level paired comparisons, while paired support reports the aggregate denominator across seeds. The OpenVLA and VLA-Adapter full-vs-perturb HR values in this representative table have aggregate paired support below 10 and are therefore annotated as low-confidence HR estimates. The OpenVLA row is at the formal denominator floor, whereas the VLA-Adapter comparison has aggregate paired support of only 4 episodes, which is below the floor; therefore, the VLA-Adapter row is treated as support-limited and is not promoted to a strong recovery-facing claim despite the visible effect direction.

**Table A5:** VLA-Adapter shield bootstrap and proxy-label support diagnostics.

Suite	Task	Bootstrap Status	Episode Support Success/Timeout	Train/Val/Calib Samples	τ	Full Mean Intervention Steps	Main Limitation	Risk-Label Source
Goal	cream_cheese	trained	76/24	6191/589/1078	0.580	25.72	–	timeout-tail fallback
Goal	bowl_top	trained	95/5	7137/822/514	3.70 × 10_−5_	18.92	–	timeout-tail fallback
Goal	stove	trained	96/4	5655/423/525	5.40 × 10^−5^	24.70	–	timeout-tail fallback
Goal	bowl_plate	failed	100/0	–	–	–	no risk-positive support	no risk-positive support
Object	bbq_sauce	trained	52/48	7779/608/704	0.045	165.80	formal gate not satisfied	timeout-tail fallback
Object	cream_cheese	trained	48/52	4806/1293/785	1.57 × 10^−5^	175.80	insufficient formal-gate support	timeout-tail fallback
Object	salad_dressing	trained	70/30	6884/1054/758	4.70 × 10^−7^	144.00	–	timeout-tail fallback
Object	tomato_sauce	trained	56/44	6860/774/696	9.30 × 10^−4^	187.60	formal gate not satisfied	timeout-tail fallback

This table reports reproducible support diagnostics for the VLA-Adapter recovery/shield branch. Episode support is computed from the shield-bootstrap source runs, and train/val/calib samples are step-level proxy-label samples. Because unsafe-attempt events are not observed in the main visual-perturbation evaluation, timeout-tail fallback labels are used for trained shields; the resulting values are therefore support diagnostics for the ROEP recovery-facing gate, not precision/recall/F1 validation of a general unsafe-action detector.

Table A5 shows that recovery/shield support is separate evidence from rollout performance. Figure A2, Figure A3 and Figure A4 show how the shield output can be inspected within a single rollout, while Table A5 reports the task-level bootstrap and intervention-support evidence behind those traces. In the Goal suite, three of four tasks have trained shieldsand receive the Recovery-facing claim supported status, while put_the_bowl_on_the_plate withholds the recovery claim because shield bootstrap fails from insufficient risk-positive support. In the Object suite, shield checkpoints exist for all four tasks, but three rows remain ecovery-facing claim withheld or Insufficient evidence because of perturbation timeout termination and formal support limitations. Mean intervention steps indicate how often the shield branch is exercised during full rollouts; they are not interpreted as recovery success rates. The proxy-label support in Table A5 also explains why classifier precision/recall/F1 is not used as a manuscript claim: under timeout-tail fallback labeling, those metrics would measure agreement with proxy terminal-risk labels rather than validation of a general unsafe-action detector.

The interpretation has three principles. First, HR decomposition is used to check whether the timeout-termination pattern claimed in the main text is actually supported by timeout evidence rather than unsafe-action spikes. Second, a nonzero delta observed in the nominal clone or perturb clone is first treated as repeat-run variability; perturbation or recovery effects are used as strong claims only when they exceed this repeat-run floor. Third, rows with large pair mismatch or low support are not promoted to main conclusions even if the effect direction is visible.

### Appendix A.4. Secondary Perturbation Severity-Sweep Support

Table A6 summarizes the secondary mild and strong perturbation profiles. The purpose of this table is not to replace the primary medium-profile comparison in Table 3, but to show how success rate and timeout termination change as perturbation severity increases. Panel A aggregates all eight tasks at the model level, while Panel B separates the Goal and Object suites.

**Table A6:** Secondary perturbation severity-sweep summary.

*Panel A: Model-level aggregate.*						
**Model**	**Mild SR**	**Strong SR**	**Strong Timeout Rate**	**Mean Clone |ΔSR|, Mild**	**Mean Clone |ΔSR|, Strong**	**Interpretation**
OpenVLA	0.762	0.068	0.932	0.060	0.005	Mild retains partial success; strong is close to timeout collapse.
X-VLA	0.998	0.320	0.680	0.008	0.060	Strong retains the highest residual success among the three models.
VLA-Adapter	0.853	0.237	0.762	0.032	0.042	Mild remains strong, but strong reveals a large Goal/Object split.
*Panel B: Model-suite aggregate.*						
**Model**	**Suite**	**Mild SR**	**Strong SR**	**Strong Timeout Rate**	**Key Pattern**	
OpenVLA	Goal	0.840	0.135	0.865	Strong success is concentrated in turn_on_the_stove.	
	Object	0.685	0.000	1.000	All four Object tasks collapse under strong perturbation.	
X-VLA	Goal	1.000	0.515	0.485	Strong Goal retains the highest residual performance.	
	Object	0.995	0.125	0.875	Object strong retains nonzero success.	
VLA-Adapter	Goal	0.945	0.475	0.525	Goal strong retains partial robustness.	
	Object	0.760	0.000	1.000	All four Object tasks collapse under strong perturbation.	

The severity sweep is secondary diagnostic evidence. It does not alter the medium-profile protocol claim status reported in Table 3.

Table A6 confirms that the strong profile acts as a severe Object-suite stress condition, while the medium profile remains the basis for the main protocol claim status.

## References

[1] Brohan A., Brown N., Carbajal J., Chebotar Y., Dabis J., Finn C., Gopalakrishnan K., Hausman K., Herzog A., Hsu J. (2022). RT-1: Robotics Transformer for Real-World Control at Scale. arXiv.

[2] Brohan A., Brown N., Carbajal J., Chebotar Y., Chen X., Choromanski K., Ding T., Driess D., Dubey A., Finn C. (2023). RT-2: Vision-Language-Action Models Transfer Web Knowledge to Robotic Control. arXiv.

[3] O’Neill A., Rehman A., Gupta A., Gupta A., Gupta A., Padalkar A., Lee A., Pooley A., Gupta A., Open X-Embodiment Collaboration (2023). Open X-Embodiment: Robotic Learning Datasets and RT-X Models. arXiv.

[4] Kim M.J., Pertsch K., Karamcheti S., Xiao T., Balakrishna A., Nair S., Rafailov R., Foster E., Lam G., Sanketi P. (2024). OpenVLA: An Open-Source Vision-Language-Action Model. arXiv.

[5] Shao X., Xu L., Zhang S., Sun G., Wu C. (2026). Fractional-Order Dynamics Learning and Control via Data-Driven Approaches: Taking Soft Manipulator as an Example. IEEE Trans. Neural Netw. Learn. Syst..

[6] Ghosh D., Walke H., Pertsch K., Black K., Mees O., Dasari S., Hejna J., Kreiman T., Xu C., Octo Model Team (2024). Octo: An Open-Source Generalist Robot Policy. arXiv.

[7] Zheng J., Li J., Wang Z., Liu D., Kang X., Feng Y., Zheng Y., Zou J., Chen Y., Zeng J. (2025). X-VLA: Soft-Prompted Transformer as Scalable Cross-Embodiment Vision-Language-Action Model. arXiv.

[8] Wang Y., Ding P., Li L., Cui C., Ge Z., Tong X., Song W., Zhao H., Zhao W., Hou P. (2025). VLA-Adapter: An Effective Paradigm for Tiny-Scale Vision-Language-Action Model. arXiv.

[9] Pumacay W., Singh I., Duan J., Krishna R., Thomason J., Fox D. THE COLOSSEUM: A Benchmark for Evaluating Generalization for Robotic Manipulation. Proceedings of the Robotics: Science and Systems 2024.

[10] Fei S., Wang S., Shi J., Dai Z., Cai J., Qian P., Li J., He X., Zhang S., Fei Z. (2025). LIBERO-Plus: In-depth Robustness Analysis of Vision-Language-Action Models. arXiv.

[11] Wang G., Zhang C., Liu Q., Zhang J., Cai J., Liu J., Liu X. (2026). LIBERO-X: Robustness Litmus for Vision-Language-Action Models. arXiv.

[12] Liu B., Zhu Y., Gao C., Feng Y., Liu Q., Zhu Y., Stone P. (2023). LIBERO: Benchmarking Knowledge Transfer for Lifelong Robot Learning. arXiv.

[13] Das M., Hussain Z., Nawaz M. (2026). Latency-Aware Benchmarking of Large Language Models for Natural-Language Robot Navigation in ROS 2. Sensors.

[14] Battiston G., Regnier R., Galibert O. (2023). Evaluation Protocol for Analogue Intelligent Medical Radars: Towards a Systematic Approach Based on Theory and a State of the Art. Sensors.

[15] Ramasubramanian A.K., Kazasidis M., Fay B., Papakostas N. (2024). On the Evaluation of Diverse Vision Systems towards Detecting Human Pose in Collaborative Robot Applications. Sensors.

[16] Liu Y., Cui X., Fan S., Wang Q., Liu Y., Sun Y., Wang G. (2024). Dynamic Validation of Calibration Accuracy and Structural Robustness of a Multi-Sensor Mobile Robot. Sensors.

[17] Martinez-Martin E., del Pobil A.P. (2019). Vision for Robust Robot Manipulation. Sensors.

[18] Mees O., Hermann L., Rosete-Beas E., Burgard W. (2022). CALVIN: A Benchmark for Language-Conditioned Policy Learning for Long-Horizon Robot Manipulation Tasks. IEEE Robot. Autom. Lett..

[19] James S., Ma Z., Arrojo D.R., Davison A.J. (2020). RLBench: The Robot Learning Benchmark and Learning Environment. IEEE Robot. Autom. Lett..

[20] Luo J., Xu C., Liu F., Tan L., Lin Z., Wu J., Abbeel P., Levine S. (2025). FMB: A Functional Manipulation Benchmark for Generalizable Robotic Learning. Int. J. Robot. Res..

[21] Guruprasad P., Sikka H., Song J., Wang Y., Liang P.P. (2024). Benchmarking Vision, Language, & Action Models on Robotic Learning Tasks. arXiv.

[22] Zhou X., Xu Y., Tie G., Chen Y., Zhang G., Chu D., Zhou P., Sun L. (2025). LIBERO-PRO: Towards Robust and Fair Evaluation of Vision-Language-Action Models Beyond Memorization. arXiv.

[23] Gu Q., Ju Y., Sun S., Gilitschenski I., Nishimura H., Itkina M., Shkurti F. (2025). SAFE: Multitask Failure Detection for Vision-Language-Action Models. arXiv.

[24] Lin Z., Duan J., Fang H., Fox D., Krishna R., Tan C., Wen B. (2025). FailSafe: Reasoning and Recovery from Failures in Vision-Language-Action Models. arXiv.

[25] Norton A., Flynn B. (2024). Towards Using Multiple Iterated, Reproduced, and Replicated Experiments with Robots (MIRRER) for Evaluation and Benchmarking. arXiv.

[26] Falco J.A., Hemphill D., Kimble K.E., Messina E.R., Norton A., Ropelato R.F., Yanco H.A. (2020). Benchmarking Protocols for Evaluating Grasp Strength, Grasp Cycle Time, Finger Strength, and Finger Repeatability of Robot End-Effectors. IEEE Robot. Autom. Lett..

[27] Ross S., Gordon G.J., Bagnell J.A. A Reduction of Imitation Learning and Structured Prediction to No-Regret Online Learning. Proceedings of the Fourteenth International Conference on Artificial Intelligence and Statistics.

[28] Belkhale S., Cui Y., Sadigh D. (2023). Data Quality in Imitation Learning. arXiv.

[29] De Ruiter A. (2025). Advanced Control Strategies for Space Systems: Integration of Model Predictive Control and Neural Networks. Advanced Control Strategies for Multi-Robot Space Systems.

